# Parent-Perceived Benefits and Harms Associated With Internet Use by Adolescent Offspring

**DOI:** 10.1001/jamanetworkopen.2023.39851

**Published:** 2023-10-26

**Authors:** Harry Graff Kimball, Francesca Fernandez, Kathleen Anne Moskowitz, Minji Kang, Lindsay M. Alexander, Kevin P. Conway, Kathleen Ries Merikangas, Giovanni Abrahão Salum, Michael Peter Milham

**Affiliations:** 1 Child Mind Institute, New York, New York; 2 Genetic Epidemiology Research Branch, Intramural Research Program, National Institute of Mental Health, Bethesda, Maryland; 3 Johns Hopkins Bloomberg School of Public Health, Baltimore, Maryland; 4 Nathan Kline Institute for Psychiatric Research, Orangeburg, New York

## Abstract

**Importance:**

Limited systematic information on familial factors and perception of the benefits and harms of internet use by youths is available. Much of the current research has been hampered by small nondiverse samples and limited information on key familial and offspring characteristics.

**Objective:**

To characterize parental perceptions and concerns about internet use associated with adolescent development, well-being, safety, family connectedness, and potential for problematic internet use.

**Design, Setting, and Participants:**

A 20-minute, English-language survey was developed with expert stakeholders using previously validated questionnaires and was conducted online between June 17 and July 5, 2022. Participants included 1005 parents of children and adolescents aged 9 to 15 years drawn from an online digital survey platform and calibrated for representation with post hoc weightings.

**Main Outcomes and Measures:**

Parent survey responses about family characteristics and internet use were used to compute Internet Addiction Test scores for parents and their offspring, Alabama Parenting Questionnaire scores, and an aggregate family connectedness score.

**Results:**

The survey cohort of 1005 parents included 568 women (56.5%) and 437 men (43.5%) with a mean age (SD) of 39.5 (6.4) years. In terms of race and ethnicity, the most common categories included Black or African American (95 [9.5%]), Latinx or Hispanic (100 [10.0%]), White (602 [59.9%]), and 2 or more races or ethnicities (122 [12.1%]). Respondents endorsed parental concerns that included exposure to harmful content (646 [64.3%]) and online bullying (533 [53.0%]). Two hundred twenty-five parents (22.4%) had specific concerns about internet addiction in their adolescent offspring, and twice as many parents reported specific concerns about internet addiction than substance addiction. However, parents also indicated that internet use improved family connectedness among immediate families (468 [46.6%]) and extended families (568 [56.5%]). Internet Addiction Test scores in adolescent offspring were correlated with parent scores (β = 0.62 [SE = 0.02]; *P* < .001) and Alabama Parenting Questionnaire-Inconsistent Discipline scores (β = 0.23 [SE = 0.11]; *P* = .04).

**Conclusions and Relevance:**

In this survey study of parent perceptions of internet use among adolescent offspring, parents believed the internet brought families closer yet also expressed concerns. Problematic internet use among youths was correlated with negative parenting styles and parent internet use. This research adds to the literature by suggesting that families, their communities, and industry may have common ground to collaborate on reducing the negative effects of internet use.

## Key Points

**Question:**

How do parents perceive the extent and consequences of internet use among their adolescent offspring, and what family characteristics and internet use patterns are associated with problematic use?

**Findings:**

This survey study of attitudes of 1005 US parents of children and adolescents aged 9 to 15 years revealed both perceived benefits (eg, family connectedness) and concerns (eg, cyberbullying, addiction) of internet use. Twice as many parents reported specific concerns about internet addiction than substance addiction.

**Meaning:**

These findings suggest that family discussions of internet use should acknowledge both perceived benefits and concerns, while correlates of problematic internet use identified in this study may inform interventions.

## Teaser

This survey study assesses parental perceptions and concerns about internet use associated with child and adolescent development, well-being, safety, family connectedness, and the potential for problematic internet use.

## Introduction

The increasing prevalence of online activities and communication, particularly during the COVID-19 pandemic, has generated concerns about potential negative consequences of internet use among youths.^1,2^ Attempts to identify young people who may be particularly vulnerable to develop excessive or problematic internet use (PIU) have been central to recent research on this topic.^3^ Excessive internet use has been associated with alcohol dependence; higher rates of depression, anxiety, and insomnia; and problems with socializing, conversation, social ease, empathic and positive feeling skills, and coping with risk.^3,4,5,6,7,8,9,10,11,12^ Other research has suggested that the negative effects of internet technologies have been overstated because much of the research has been based on small selected samples without inclusion of information on potential correlates of internet use and negative outcomes.^13,14,15,16^

Parent perceptions are important to quantify youth internet use, as youth-reported research is more difficult to undertake. Studies have shown acceptable levels of agreement between parent and offspring reports on media use by youths.^17,18^ In addition, parental perception and regulation of youth internet activities have been identified as important correlates of youth internet behavior.^19,20^ Parent perceptions of family interactions, including parenting and sibling relationships, have previously been linked to child and adolescent PIU in Chinese^21^ and Japanese populations,^22^ but to our knowledge, similar research has not been undertaken in a large sample of US families. Families with a higher negative perception of the internet were most likely to have intergenerational conflict.^23^

The primary goal of the present study was to characterize parental perceptions and concerns about internet use associated with child and adolescent development, well-being, safety, family connectedness, and potential for PIU. This was accomplished through the conduct of an online survey of parents of offspring aged 9 to 15 years (referred to hereinafter as *adolescents*) to obtain a broad sample with respect to demographic and socioeconomic status. The survey assessed parents’ perception of the risks and benefits of internet use in different domains, including (1) development (ie, socioemotional, cognitive, physical), safety (ie, bullying, content exposure), and well-being; (2) potential for addiction; and (3) family connectedness. The design and implementation of the survey were based on the previous Coronavirus Health and Impact Survey,^24,25^ which demonstrated associations between COVID-19–related stress and adverse mental health outcomes.

## Methods

### Survey Design

Because all data were collected anonymously, no Institutional Review Board (IRB) oversight was required. Exemption from oversight was approved by the Advarra IRB. Participants using the Prolific website were required to agree to the Terms of Service notification before being allowed to complete surveys. Participants in the Ipsos survey had to agree to standard terms to be a part of the partner panel and to Ipsos’ terms during the survey. Per the IRB exemption, no additional informed consent was required. The survey administration and reporting conformed to the American Association for Public Opinion Research (AAPOR) reporting guideline.

The 20-minute online survey consisted of 181 questions in English. The survey instrument was developed in consultation with stakeholders who included psychiatrists, psychologists, and epidemiologists. The survey included measures derived from a series of validated questionnaires. From the Coronavirus Health and Impact Survey,^25^ measures of demographics, sleep, exercise, media use, and mood circumplex were used. The entirety of the Internet Addiction Test (IAT) short version^26^ was included to calculate IAT scores and assess PIU. The Alabama Parent Questionnaire short form^27^ was similarly included in its entirety and used to derive measures of positive parenting, inconsistent discipline, and poor supervision. Five items from the 13-item Parental Control Scale parent version^28^ were used to assess parent perception of behavioral control over their adolescent offspring. The entirety of the 2019 Parent Mediation Items^29^ was used to derive a measure of parental mediation of offspring internet use. Portions of EU Kids Online,^30^ a large survey of online behavior, were used to assess internet usage. Portions of the Family Adaptability and Cohesion Evaluation Scale^31^ were used to create a measure of family connectedness. Parts of the Child Parent Relationship Scale^32^ were used to assess perception of offspring dependence. Portions of the Patient Reported Outcomes Measurement Information System Peer Relationship Scale Short Form were included as a measure for offspring likability, peer friendships, and social reputation.^33^

Internet use was defined as connected computers and web browsing; email and other messaging; mobile phones (whether used for phone calls, messaging, social media, or accessing streaming media or gaming); connected devices such as portable game consoles; digital media; streaming video; internet TV; and gaming consoles connected to online communities. An overview of elements included from the questionnaires is found in the eAppendix in Supplement 1.

### Recruitment

The survey was piloted on the Prolific website using samples in the US (307 participants; offspring aged 5-21 years) and the United Kingdom (693 participants; offspring aged 5-21 years) previously established to study COVID-19 impact on mental health outcomes.^24,25^ Participation in the pilot survey was limited to the first 1000 respondents.

Following the pilot survey, the full survey was fielded with families and parents of offspring aged 9.00 to 15.99 years by Ipsos^34^ using a sampling strategy designed to enable US national representativeness. The age range of 9 to 15 years was chosen to include 2 distinct groups for comparison: a younger group in latency (aged 9.00-11.99 years) and an early adolescent group (aged 12.00-15.99 years). Ipsos performed all survey administration activities. The sample was randomly drawn from Ipsos’ partner online panel source, m360 Research.^35^ Ipsos contacted panel members via the online survey platform, email, and/or telephone; participants completed the survey online. Respondent characteristics were calibrated to be representative of the US Census 2019 American Community Survey. The sample reflected fixed targets on demographic characteristics, and recruitment continued until these targets were achieved. Participants were asked to identify their own gender identity as well as their child's gender identity (per their knowledge) from a list of preset options including male, female, nonbinary, transgender male, transgender female, other, and prefer not to answer. Participants were also asked which race or ethnicity category best described them and (separately) their offspring from a list of preset options, including White; Black or African American; Latino/Latina/Latinx or Hispanic; American Indian or Alaska Native; East Asian or Pacific Islander; South or Southeast Asian; Middle Eastern or North African; Caribbean; a race, ethnicity, or origin not listed; and prefer not to answer. Race and ethnicity data were included in the study to control for confounding and to explore disparities in internet impact on youth and families. Post hoc weights were made to reflect population characteristics on gender identity, age, race and ethnicity, region, and educational level. Respondents agreed to Ipsos’s terms before the survey.

Parents completed the survey on their own behalf and according to their perception of their offspring’s use of digital technologies; survey outcomes were based only on parent reports. Questions were completed using a Likert scale from 1 (strongly disagree) to 5 (strongly agree). The survey was conducted between June 17 and July 5, 2022.

### Statistical Analysis

Demographic and descriptive statistics were calculated. Group differences between latency and early adolescence were assessed by stratifying by age (9.00-11.99 years and 12.00-15.99 years) and comparing key variables across strata with a Welch 2-sample *t* test. Multiple general linear models examined correlates of offspring internet addiction, parental internet addiction, and associations between parental and offspring internet addiction and demographic and parenting factors. Analyses also explored internet use across the following 7 content categories: general content (eg, web browsing, shopping news, podcast, music), video streaming (eg, YouTube, Twitch, Netflix, digital television), social networking (eg, Facebook), gaming (eg, Among Us, Call of Duty, Fortnite, Grand Theft Auto, Minecraft, Nintendo Switch, Roblox), content sharing (eg, TikTok, Instagram, Triller, VSCO), messaging (eg, Reddit, WhatsApp, Snapchat, Discord, Telegram, Messenger, Kik, Marco Polo, Hangouts), and immersive environments (eg, Houseparty, Gathertown, Second Life, Nowhere). To ensure a normal distribution of residuals in the general linear models, outliers with a Cook’s distance greater than 3 times the mean were identified and discarded. Normality was evaluated with results of a Kolomogrov-Smirnov test.

Parallel analysis investigated the number of factors that best characterize family connectedness, parent mediation regarding internet use, and coparenting style. Parallel analysis suggested that a single factor was most suitable for family connectedness, 3 factors for parent mediation (positive, restrictive, and technical), and 2 factors for coparenting questions (positive and negative perceptions of the coparent). Confirmatory factor analysis assessed fit of a theoretical model using root mean square error of approximation, comparative fit index, Tucker-Lewis Index, and standardized root mean squared residual (eMethods in Supplement 1).

Based on the number of parallel statistical tests in the internet use analysis, a Bonferroni correction 2-sided α < .01 was calculated. Results were assessed according to this significance threshold. Data were analyzed using R, version 4.3.1X (R Project for Statistical Computing).

## Results

The final survey cohort included 1005 participants consisting of 568 women (56.5%) and 437 men (43.5%) with a mean (SD) age of 39.5 (6.4) years. A total of 1355 participants enrolled and consented to participate, and 350 dropped out.

### Demographic Characteristics

Respondent demographics are shown in Table 1 alongside those from the Prolific data pilot (N = 995). Demographics of both the pilot and full survey cohorts were comparable. In the Ipsos cohort, there were more female than male parents (568 [56.5%] vs 437 [43.5%]) but more male than female adolescent offspring (583 [58.0%] vs 416 [41.4%]). Parent race and ethnicity categories included American Indian or Alaska Native (10 [1.0%]); Black or African American (95 [9.5%]); Caribbean (5 [0.5%]); East Asian, Native Hawaiian, or Other Pacific Islander (42 [4.2%]); Latinx or Hispanic (100 [10.0%]); Middle Eastern or North African (4 [0.4%]); South or Southeast Asian (21 [2.1%]); White (602 [59.9%]); 2 or more races or ethnicities (122 [12.1%]); other race or ethnicity (including respondents who answered “a race, ethnicity, or origin not listed” to the question “Which best describes you/your child?”) (4 [0.4%]); and those who preferred not to answer (2 [0.2%]). Most parents were married (701 [69.8%]) and had between 1 and 3 offspring (865 [86.1%]). Educational levels were evenly represented across the sample and household income was rated as being $50 000 to $149 999 for more than one-half of respondents (539 [53.6%]), with a further 271 (27.0%) earning $49 999 or less and 185 (18.4%) earning $150 000 or more.

**Table 1.** Demographic Characteristics of Survey Populations From the Ipsos Cohort and the Pilot Prolific Cohort

Characteristic	No. (%) of respondents	
	Ipsos sample (N = 1005)	Prolific sample (N = 995)
Gender		
Parents		
Female	568 (56.5)	695 (69.8)
Male	437 (43.5)	291 (29.2)
Other	0	8 (0.8)
Prefer not to answer	0	1 (0.1)
Offspring		
Female	416 (41.4)	464 (46.6)
Male	583 (58.0)	510 (51.3)
Other	4 (0.4)	17 (1.7)
Prefer not to answer	2 (0.2)	4 (0.4)
Age, y		
Parents		
20-29	39 (3.9)	31 (3.1)
30-39	494 (49.2)	408 (41.0)
40-49	389 (38.7)	423 (42.5)
50-59	83 (8.3)	122 (12.3)
60-69	0	9 (0.9)
Missing	0	2 (0.2)
Offspring		
≤5	0	3 (0.3)
6-10	212 (21.1)	330 (33.2)
11-15	793 (78.9)	395 (39.7)
16-20	0	247 (24.8)
≥21	0	20 (2.0)
Race and ethnicity		
Parents		
American Indian or Alaska Native	10 (1.0)	2 (0.2)
Black or African American	95 (9.5)	42 (4.2)
Caribbean	5 (0.5)	6 (0.6)
East Asian, Native Hawaiian, or Other Pacific Islander	42 (4.2)	15 (1.5)
Latinx or Hispanic	100 (10.0)	13 (1.3)
Middle Eastern or North African	2 (0.2)	2 (0.2)
South or Southeast Asian	21 (2.1)	26 (2.6)
White	602 (59.9)	849 (85.3)
≥2	122 (12.1)	26 (2.6)
Other^a^	4 (0.4)	11 (1.1)
Prefer not to answer	2 (0.2)	3 (0.3)
Offspring		
American Indian or Alaska Native	8 (0.8)	1 (0.1)
Black or African American	97 (9.7)	35 (3.5)
Caribbean	2 (0.2)	4 (0.4)
East Asian, Native Hawaiian, or Other Pacific Islander	33 (3.3)	10 (1.0)
Latinx or Hispanic	92 (9.2)	10 (1.0)
Middle Eastern or North African	1 (0.1)	2 (0.2)
South or Southeast Asian	21 (2.1)	23 (2.3)
White	580 (57.7)	805 (80.9)
≥2	163 (16.2)	73 (7.3)
Other^a^	5 (0.5)	25 (2.5)
Prefer not to answer	3 (0.3)	7 (0.7)
Caregiver relationship		
Father	416 (41.4)	285 (28.6)
Mother	559 (55.6)	699 (70.3)
Other	30 (3.0)	11 (1.1)
Marital status		
Married	701 (69.8)	615 (61.8)
Domestic partnership	71 (7.1)	150 (15.1)
Separated	38 (3.8)	29 (2.9)
Divorced	72 (7.2)	73 (7.3)
Never married	115 (11.4)	115 (11.6)
Widowed	8 (0.8)	3 (0.3)
Single caregiver		
No	573 (57.0)	773 (77.7)
Yes	432 (43.0)	219 (22.0)
Missing	0	3 (0.3)
Highest parental educational level		
High school diploma or below	183 (18.2)	130 (13.1)
Some college or 2-y degree	239 (23.8)	247 (24.8)
4-y College or some school beyond college	298 (29.7)	301 (30.3)
Graduate program or professional degree	285 (28.4)	317 (31.9)
Household income, $		
≤24 999	109 (10.8)	123 (12.4)
25 000-49 999	162 (16.1)	283 (28.4)
50 000-99 999	288 (28.7)	359 (36.1)
100 000-149 999	251 (25)	129 (14)
≥150 000	185 (18.4)	58 (5.8)
Prefer not to answer	10 (1)	43 (4.3)
Family location		
Large metropolitan area	293 (29.2)	135 (13.6)
Metropolitan area	129 (12.8)	96 (9.6)
Medium-sized metropolitan area	121 (12.0)	166 (16.7)
Small metropolitan area	61 (6.1)	131 (13.2)
Large suburban area	162 (16.1)	155 (15.6)
Small suburban area	115 (11.4)	209 (21.0)
Rural area	124 (12.3)	103 (10.4)
No. of offspring		
0	0	1 (0.1)
1	236 (23.5)	282 (28.3)
2	403 (40.1)	487 (48.9)
3	226 (22.5)	153 (15.4)
4	91 (9.1)	54 (5.4)
≥5	49 (4.9)	18 (1.8)
No. of people in the household		
1-2	69 (6.9)	100 (10.1)
3-4	588 (58.5)	691 (69.4)
5-6	306 (30.4)	183 (18.4)
7-8	34 (3.4)	17 (1.7)
≥9	8 (0.8)	4 (0.4)

^a^ Includes respondents who answered “a race, ethnicity, or origin not listed” to the question “Which best describes you/your child?”

### Parental Attitudes and Concerns About Internet Use

Most parents reported confidence that their offspring can use the internet responsibly (720 [71.6%]) and that they can discuss internet use with their offspring (812 [80.8%]). Similarly, parents were confident about gauging appropriate durations of screen time for their offspring (720 [71.6%]) and having strategies to manage screen time with healthy alternatives (704 [70.0%]). Overall, most parents were confident that their offspring received effective instruction on safe internet use (656 [65.3%]).

**Figure 1.**
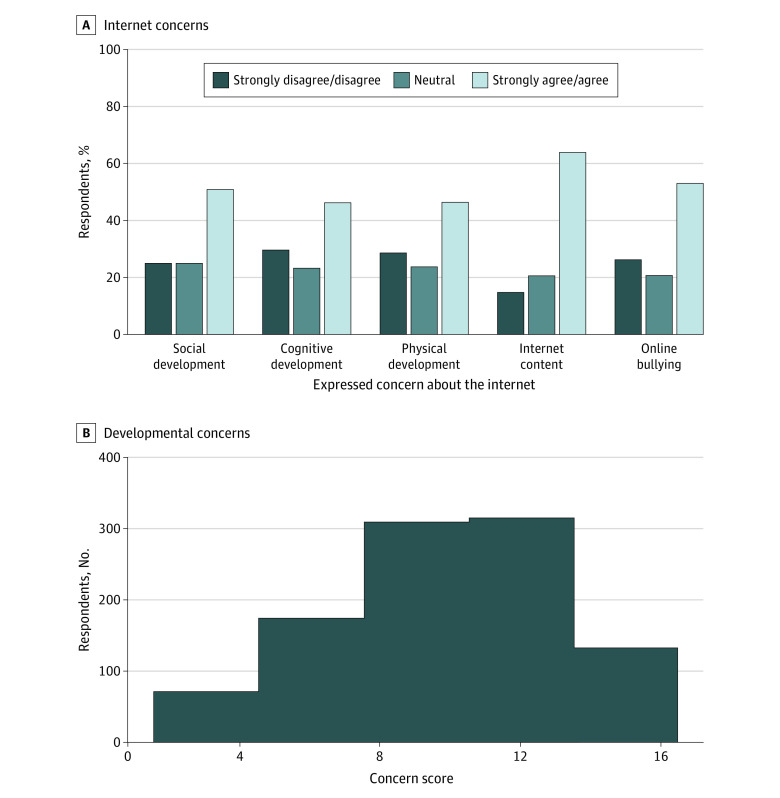
Parent Concerns About the Effect of the Internet on Their Offspring The developmental concern score is calculated as the total concern scores for social, cognitive, and physical development, each with a possible score of 5 (15 total). Higher scores indicate greater concern.

Nevertheless, respondents expressed concerns about the internet and its effect on their offspring’s development. As shown in Figure 1A, approximately one-half of parents expressed specific concerns about internet use on their offspring’s social (514 [51.1%]), cognitive 464 [46.2%]), and physical development (469 [46.7%]). More than one-half of parents expressed concerns about access to inappropriate content (646 [64.3%]) and cyberbullying (533 [53.0%]). Figure 1B shows the distribution of the total developmental concern score reported by parents. The mean (SD) score was 9.78 (3.20) of 15 possible points (5 per domain) confirmed by factor analysis to be single constructs. Further, more than one-half of respondents believe that both the government (520 [51.7%]) and technology suppliers (642 [63.9%]) should be more proactive in regulating internet content and internet use.

**Figure 2.**
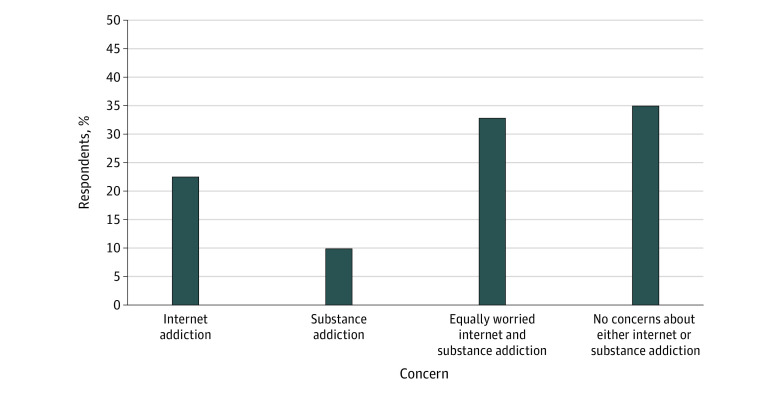
Parent Concerns About Internet and Substance Addiction in Their Offspring

Concerns about internet addiction were contrasted with concerns about substance addiction (Figure 2). Three hundred fifty-one respondents (34.9%) had no concerns about internet or substance addiction, and 331 (32.9%) were equally worried about internet and substance addiction. Of the remainder, more than twice as many parents (225 [22.4%]) were solely concerned with internet addiction than with substance addiction (98 [9.8%]). When questioned about specific internet activities and respondents’ perceived risk for internet addiction, social networking programs were highlighted as being most concerning (304 [30.2%]), followed by video gaming (124 [12.3%]). Almost one-fifth of parents (187 [18.6%]) reported no concerns with specific internet platforms (eFigure 1 in Supplement 1). In the Prolific pilot data set, the same question was asked but with greater granularity. For social networking, Facebook was considered the platform of most concern, while among those who considered gaming to be the highest concern (n = 71), Fortnite was considered most concerning (16 [22.5%]) followed by Roblox (12 [16.9%]) and Grand Theft Auto (11 [15.5%]).

### IAT and PIU

#### Description of IAT

Parent scores on the IAT were largely below the threshold for problematic use (≤39); 596 (59.3%) scored below this threshold.^36,37^ However, parents’ evaluation of their offspring’s IAT score had a more even spread of responses, with scores ranging from less than 10 to 50, suggesting that parents observed a greater extent of PIU in their offspring (eFigure 2 in Supplement 1). A higher proportion of parents reported IAT scores greater than 70 compared with offspring, suggesting severe problematic use, but this finding was not significant.

#### Correlates of Child IAT Scores

##### Parent IAT

Table 2 presents the results from the general linear model analysis that examined the association between parent IAT scores and offspring IAT scores (the dependent variable) after adjusting for demographic factors, parent IAT scores, and parenting factors. Parental IAT scores were positively correlated with offspring IAT scores (β = 0.62 [SE = 0.02]; *P* < .001), even after accounting for effects of offspring age and gender, parental educational level, and parenting factors.

**Table 2.** General Linear Model of Offspring IAT Scores Associated With Parent IAT Scores, Parent APQ-SF Scores, and Parenting Domain Factors

Variable	No. of respondents	β (SE)	*P* value
Intercept	NA	3.69 (2.59)	.16
Parent IAT score	NA	0.62 (0.02)	<.001
APQ-SF domain			
Positive parenting	NA	0.16 (0.10)	.12
Inconsistent discipline	NA	0.23 (0.11)	.04
Poor supervision	NA	0.20 (0.11)	.07
Parental mediation of internet use			
Parent mediation factor 1 (positive)	NA	−0.18 (0.12)	.14
Parent mediation factor 2 (restrictive)	NA	0.19 (0.14)	.17
Parent mediation factor 3 (technical)	NA	0.17 (0.10)	.08
Coparenting factor 1 (positive)	NA	−0.12 (0.05)	.02
Coparenting factor 2 (negative)	NA	0.16 (0.04)	<.001
Parent communication score	NA	0.08 (0.08)	.31
Parent control score	NA	0.01 (0.09)	.88
Offspring age	NA	0.17 (0.13)	.20
Offspring gender			
Female (reference)	NA	NA	NA
Male	543	0.46 (0.51)	.36
Other	1	5.79 (7.62)	.45
Highest parental educational level			
High school or below (reference)	166	NA	NA
Some college or 2-y degree	220	−2.31 (0.79)	<.001
4-y College or some graduate	278	−1.56 (0.86)	.07
Graduate or professional degree	273	−1.52 (0.96)	.11
Household income, $			
<25 000 (Reference)	96	NA	NA
25 000-49 999	148	0.80 (1.08)	.46
50 000-99 999	270	1.01 (1.16)	.38
100 000-149 999	241	−0.90 (1.00)	.37
≥150 000	182	1.42 (0.96)	.14

Abbreviations: APQ-SF, Alabama Parent Questionnaire–Short Form; IAT, Internet Addiction Test; NA, not applicable.

##### Parenting Style

The distribution of scores by the 3 parenting dimensions measured by the Alabama Parent Questionnaire–Short Form—Positive Parenting, Inconsistent Discipline, and Poor Monitoring and Supervision—appear in eFigure 3 in Supplement 1. In a multivariable general linear model that did not include parent IAT scores, there were associations between offspring IAT scores and inconsistent discipline (β = 0.85 [SE = 0.17]; *P* < .001), poor monitoring and supervision (β = 0.90 [SE = 0.16]; *P* < .001), parent mediation (β = 0.86 [SE = 0.21]; *P* < .001), positive perception of coparent (factor 1) (β = −0.24 [SE = 0.07]; *P* = .001), and negative perception of coparent (factor 2) (β = 0.28 [SE = 0.06]; *P* < .001). In the final adjusted model (Table 2), there were associations between offspring IAT scores and inconsistent discipline (β = 0.23 [SE = 0.11]; *P* = .04), coparenting factor 1 (β = -0.12 [SE = 0.05]; *P* = .02), and coparenting factor 2 (β = 0.16 [SE = 0.04]; *P* < .001).

#### Internet Use Category

Table 3 presents the results from the general linear model analysis that examined the association between internet use category and offspring IAT scores (the dependent variable) after adjusting for demographic factors. Adolescent offspring IAT scores were associated with the offspring’s use of immersive environments such as virtual or augmented reality platforms for 3 to 6 hours daily (β = 8.97 [SE = 1.83]; *P* < .001) and over 6 hours daily (β = 7.17 [SE = 2.15]; *P* < .001), suggesting an elevated risk of addiction for these online activities. Offspring IAT scores were also associated with all levels of parental streaming video use, including under 1 hour (β = 6.70 [SE = 2.17]; *P* < .001), 1 to less than 3 hours (β = 8.72 [SE = 2.09]; *P* < .001), 3 to 6 hours (β = 9.13 [SE = 2.21]; *P* < .001), and over 6 hours daily (β = 7.16 [SE = 2.45]; *P* < .001); 3 to 6 hours of parent gaming daily (β = 4.00 [SE = 1.61]; *P* = .01); and different levels of immersive environments, including 1 to less than 3 hours (β = 4.77 [SE = 1.37]; *P* < .001) and 3 to 6 hours daily (β = 7.05 [SE = 1.90]; *P* < .001). Parent use of immersive environments was also associated with parent IAT scores for 1 to less than 3 hours (β = 6.73 [SE = 1.69]; *P* < .001) and 3 to 6 hours daily (β = 11.87 [SE = 2.38]; *P* < .001) and with family impact for under 1 hour (β = 2.43 [SE = 0.79]; *P* < .001), 1 to less than 3 hours (β = 2.93 [SE = 0.81]; *P* < .001), 3 to 6 hours (β = 4.06 [SE = 1.12]; *P* < .001), and over 6 hours daily (β = 6.89 [SE = 1.56]; *P* < .001). This analysis also demonstrated an association between total family impact score and offspring use of social networking for 1 to less than 3 hours (β = 2.96 [SE = 0.92]; *P* < .001), 3 to 6 hours (β = 4.66 [SE = 1.15]; *P* < .001), and over 6 hours daily (β = 5.24 [SE = 1.36]; *P* < .001).

**Table 3.** Offspring and Parent Internet Use Category Associated With Parent and Adolescent Offspring IAT Scores and Family Impact Score

Dependent variable	Child use				Parent use						No. of offspring^b^	No. of parents^b^
	Family Impact Score		Child IAT score		Family Impact Score		Child IAT score		Parent IAT score			
	β (SE)	*P* value^a^	β (SE)	*P* value^a^	β (SE)	*P* value^a^	β (SE)	*P* value^a^	β (SE)	*P* value^a^		
Intercept	38.31 (3.19)	<.001	29.57 (5.40)	<.001	35.31 (2.40)	<.001	24.27 (3.92)	<.001	27.78 (4.92)	<.001	NA	NA
Time spent on internet by use type, h												
General internet												
0 (Reference)	NA	NA	NA	NA	NA	NA	NA	NA	NA	NA	81	43
<1	−0.3 (1.0)	.76	−2.57 (1.72)	.13	4.02 (1.46)	.01	−2.12 (2.30)	.36	−2.78 (2.93)	.34	243	248
1 to <3	0.36 (1.02)	.73	−2.07 (1.75)	.24	4.39 (1.43)	<.001	−0.74 (2.26)	.74	−2.02 (2.87)	.48	292	382
3 to 6	0.01 (1.22)	.99	−0.67 (2.03)	.74	2.96 (1.54)	.05	−2.21 (2.43)	.36	−4.80 (3.13)	.13	162	173
>6	0.50 (1.40)	.72	−0.95 (2.42)	.69	4.49 (1.72)	.01	−3.38 (2.70)	.21	−7.15 (3.51)	.04	90	97
Streaming videos												
0 (Reference)	NA	NA	NA	NA	NA	NA	NA	NA	NA	NA	12	44
<1	4.01 (2.36)	.09	4.49 (3.92)	.25	3.08 (1.30)	.02	6.70 (2.17)	<.001	8.83 (2.74)	<.001	104	189
1 to <3	4.14 (2.27)	.07	4.26 (3.80)	.26	3.53 (1.25)	<.001	8.72 (2.09)	<.001	9.46 (2.63)	<.001	355	384
3 to 6	4.74 (2.32)	.04	5.70 (3.88)	.14	4.16 (1.33)	<.001	9.13 (2.21)	<.001	10.09 (2.80)	<.001	217	213
>6	4.53 (2.39)	.06	8.16 (3.99)	.04	3.24 (1.48)	.03	7.16 (2.45)	<.001	7.32 (3.12)	.02	180	113
Social networking												
0 (Reference)	NA	NA	NA	NA	NA	NA	NA	NA	NA	NA	237	80
<1	0.31 (0.89)	.72	−1.28 (1.50)	.39	−1.87 (1.08)	.08	−1.39 (1.83)	.45	1.67 (2.35)	.48	176	282
1 to <3	2.96 (0.92)	<.001	1.10 (1.57)	.48	−0.39 (1.11)	.72	−0.11 (1.86)	.95	4.45 (2.39)	.06	226	316
3 to 6	4.66 (1.15)	<.001	2.77 (1.89)	.14	−1.14 (1.21)	.35	0.18 (2.01)	.93	3.44 (2.59)	.18	130	166
>6	5.24 (1.36)	<.001	−0.02 (2.27)	.99	−1.08 (1.47)	.46	1.02 (2.39)	.67	3.20 (3.06)	.30	99	99
Gaming												
0 (Reference)	NA	NA	NA	NA	NA	NA	NA	NA	NA	NA	47	268
<1	3.35 (1.27)	.01	1.51 (2.22)	.50	1.18 (0.77)	.12	1.43 (1.27)	.26	−2.54 (1.62)	.12	143	205
1 to <3	2.07 (1.16)	.08	1.27 (2.06)	.54	1.59 (0.75)	.03	2.62 (1.27)	.04	0.24 (1.59)	.88	312	279
3 to 6	−0.03 (1.25)	.98	1.52 (2.19)	.49	2.65 (0.98)	.01	4.00 (1.61)	.01	1.54 (2.03)	.45	192	127
>6	0.11 (1.36)	.94	−0.38 (2.36)	.87	0.70 (1.34)	.60	2.92 (2.23)	.19	−0.62 (2.79)	.82	174	64
Content sharing												
0 (Reference)	NA	NA	NA	NA	NA	NA	NA	NA	NA	NA	167	192
<1	−0.63 (0.94)	.50	2.04 (1.61)	.21	2.99 (0.77)	<.001	−1.20 (1.29)	.35	−1.29 (1.64)	.43	176	284
1 to <3	−0.44 (0.94)	.64	2.07 (1.59)	.19	1.36 (0.85)	.11	−2.26 (1.42)	.11	−2.85 (1.79)	.11	281	266
3 to 6	−3.59 (1.17)	<.001	−0.04 (1.97)	.99	1.66 (1.04)	.11	−0.12 (1.71)	.94	1.90 (2.18)	.38	138	131
>6	−2.79 (1.35)	.04	−0.51 (2.28)	.82	1.95 (1.37)	.15	1.83 (2.27)	.42	2.83 (2.90)	.33	106	70
Messaging and/or forums												
0 (Reference)	NA	NA	NA	NA	NA	NA	NA	NA	NA	NA	201	176
<1	0.86 (0.87)	.33	0.17 (1.49)	.91	−0.40 (0.80)	.62	1.55 (1.34)	.25	2.29 (1.69)	.18	205	296
1 to >3	0.14 (0.95)	.88	−0.68 (1.61)	.68	0.79 (0.88)	.37	0.44 (1.44)	.76	−0.63 (1.83)	.73	233	268
3 to 6	1.95 (1.18)	.10	−0.64 (1.97)	.75	0.62 (1.04)	.55	2.88 (1.71)	.09	1.46 (2.17)	.50	125	139
>6	2.20 (1.38)	.11	0.13 (2.31)	.95	0.04 (1.42)	.98	1.07 (2.35)	.65	2.13 (2.93)	.47	104	64
Immersive environments												
0 (Reference)	NA	NA	NA	NA	NA	NA	NA	NA	NA	NA	409	486
<1	2.47 (0.80)	<.001	0.43 (1.36)	.75	2.43 (0.79)	<.001	0.65 (1.32)	.62	0.27 (1.66)	.87	153	160
1 to <3	1.96 (0.84)	.02	2.93 (1.43)	.04	2.93 (0.81)	<.001	4.77 (1.37)	<.001	6.73 (1.69)	<.001	153	184
3 to 6	4.61 (1.07)	<.001	8.97 (1.83)	<.001	4.06 (1.12)	<.001	7.05 (1.90)	<.001	11.87 (2.38)	<.001	85	77
>6	4.25 (1.27)	<.001	7.17 (2.15)	<.001	6.89 (1.56)	<.001	7.11 (2.63)	.01	7.54 (3.33)	.02	68	36
Child age	−0.61 (0.15)	<.001	−0.11 (0.25)	.66	−0.38 (0.13)	<.001	0.18 (0.22)	.42	−0.21 (0.28)	.45	NA	NA
Child gender												
Female (reference)	NA	NA	NA	NA	NA	NA	NA	NA	NA	NA	NA	NA
Male	0.01 (0.54)	.99	1.45 (0.91)	.11	−0.19 (0.50)	.70	0.89 (0.84)	.29	0.34 (1.06)	.75	NA	NA
Other	−3.12 (8.47)	.71	−1.21 (12.99)	.93	NA	NA	4.17 (12.79)	.74	5.27 (15.89)	.74	NA	NA
Prefer not to answer	NA	NA	−14.13 (15.87)	.37	NA	NA	NA	NA	−9.06 (13.32)	.50	NA	NA
Highest parental educational level												
High school or below (reference)	NA	NA	NA	NA	NA	NA	NA	NA	NA	NA	NA	NA
Some college or 2-y degree	−2.47 (0.83)	<.001	−3.17 (1.43)	.03	−3.39 (0.79)	<.001	−4.24 (1.32)	<.001	−2.79 (1.66)	.09	NA	NA
4-y College or some graduate	−2.69 (0.89)	<.001	−5.18 (1.54)	<.001	−3.16 (0.84)	<.001	−5.28 (1.40)	<.001	−7.19 (1.76)	<.001	NA	NA
Graduate or professional degree	−0.74 (0.98)	.45	−2.71 (1.70)	.11	−0.59 (0.94)	.53	−3.01 (1.57)	.06	−4.15 (1.95)	.03	NA	NA
Household income, $												
<25 000 (Reference)	NA	NA	NA	NA	NA	NA	NA	NA	NA	NA	NA	NA
25 000-49 999	−1.67 (1.03)	.10	−2.27 (1.77)	.20	−3.01 (0.97)	<.001	−0.17 (1.63)	.92	−2.76 (2.05)	.18	NA	NA
50 000-99 999	−0.73 (0.97)	.45	0 (1.70)	>.99	−2.02 (0.91)	.03	1.14 (1.56)	.46	−0.12 (1.94)	.95	NA	NA
100 000-149 999	0.20 (1.10)	.85	1.31 (1.91)	.49	−1.74 (1.03)	.09	1.49 (1.75)	.39	2.41 (2.20)	.27	NA	NA
≥150 000	0.56 (1.18)	.63	2.37 (2.06)	.25	−1.10 (1.13)	.33	1.89 (1.90)	.32	2.49 (2.38)	.30	NA	NA
Prefer not to answer	4.14 (3.88)	.29	−2.20 (9.23)	.81	1.37 (4.38)	.75	11.77 (6.44)	.07	2.65 (7.27)	.72	NA	NA

Abbreviations: IAT, Internet Addiction Test; NA, not applicable.

^a^ Bonferroni correction of α < .01.

^b^ Offspring and parent numbers for different internet use types and levels of daily use included to assist in interpretation.

### Correlates of Family Connectedness

When asked for perceptions of the effect of internet use on family connectedness, respondents were generally in agreement that its use increases family bonds (eFigure 4 in Supplement 1). This perceived benefit was marginally greater among immediate families vs extended families (568 [56.5%] vs 468 [46.6%]). However, up to one-fifth of respondents indicated that the internet did not improve family connectedness (210 [20.9%]) and up to one-third remained neutral (327 [32.5%]).

Other perceived family benefits of internet use were reported (eFigure 5 in Supplement 1), including feeling able to share positive experiences (653 [65.0%] rated as beneficial), improved quality of communication (613 [61.0%]), increased ability to be flexible (600 [59.7%]), and more time spent together as a family (585 [58.2%]). The mean (SD) family impact score across the 10 question domains was 37.2 (8.9), indicative of a positive perception of the internet for connecting families (eFigure 5 in Supplement 1).

### Time Spent Using the Internet and Age Differences

Most parents believe that their adolescent offspring engage in fewer than 3 hours of screen time per day and a smaller proportion spend 4 hours or more (eFigure 6 in Supplement 1). This was consistent with pilot data from the Prolific cohort; these data also showed an increase in time spent on the internet during the early period of the COVID-19 pandemic, but with a return to pre–COVID-19 levels largely by summer of 2022. There were no statistically significant differences between the 2 age groups on offspring IAT score (*t*_600_ = −0.54; *P* = .59) and family impact score (*t*_604_ = 0.69; *P* = .49).

## Discussion

These survey data confirm that the use of digital technology in 2022 has mixed consequences for families.^38,39^ Parents indicated that internet use improved the sense of family connectedness through an ability to share positive experiences, an increased sense of family closeness, an increased ability for family flexibility, and positive benefits on family time spent together. The association of social networking platform use in youths with family impact scores suggest that enhanced within-family network communication may be a benefit of internet use.

Perceived benefits of internet use were balanced by parental concerns about the potential of social networking platforms to contribute to internet addiction, echoing recent warnings by the US Surgeon General.^40^ Parents also endorsed overriding concerns about the risks for cyberbullying and exposure to inappropriate or harmful content. Previous work found that these dangers are particularly relevant to specific age and social contextual factors.^41,42^ The benefit-risk balance was particularly evident for immersive virtual reality technologies, which were simultaneously perceived as having a positive effect on family connectedness and increasing the risk of PIU. This association also revealed an important trend in parent use. The association of parent virtual reality use with both parent and offspring PIU levels and with positive family impact scores could result from early adopters gravitating toward this new technology (ie, tech-savvy parents with positive perceptions of the internet who nonetheless use the internet in ways that elevate their IAT scores). Overall, parents did not view immersive platforms as an area of concern, suggesting that the risks of this new technology (real and perceived) are still emerging.

Importantly, these data suggest that PIU in young people might reflect PIU in their parents. In this study, problematic internet use patterns among parents were associated with problematic patterns in their adolescent offspring, even after considering the effects of negative parenting practices like inconsistent discipline and poor supervision. This is consistent with previous findings wherein adolescent internet use and PIU was influenced by parental use of internet and digital resources with a significant relationship between parent and offspring use.^43^ While not a primary consideration for this survey, comparisons of parenting style demonstrated some correlation with high IAT scores in young people. These data warrant further investigation but support earlier literature^44,45,46,47^ suggesting the association of parental mental health, parenting style, and familial demographics with development of PIU.

### Strengths and Limitations

The large sample with broad racial, ethnic, and geographic representation enhances the generalizability of these findings to the US. Additionally, the inclusion of important parental, youth, and familial factors that may influence internet use enabled us to examine the correlates of specific patterns of internet use that may distinguish the factors associated with the perceived benefits and harms of internet use. Finally, our focus on familial factors highlights the importance of the family unit in interpreting correlates of internet use, as well as the role of the family in modifying negative use and prevention of harmful use and addiction.

This study has some limitations. This survey did not include youth self-report on internet use and its correlates. Parent reports may not accurately reflect youth perceptions. Nevertheless, work in this field is beginning to quantify patterns of parent-youth agreement on internet use and its consequences.^21^

## Conclusions

In this survey study, parents appeared to appreciate the positive familial consequences of internet connectivity, a perspective that is borne out by the correlates of family connectedness explored in this study. They were also deeply concerned about the risks of cyberbullying, damaging content, and internet addiction among their offspring. Crucially, this research strengthens and extends the literature on correlates of PIU among adolescents, including parenting styles and parental internet use. The salient perception of enhanced familial interconnectedness afforded by internet use in this study should be leveraged into family interventions designed to enhance open communication and monitoring of potential dangers of internet use in youth.

## Notes

Supplement 1. **eAppendix.** Survey Questionnaire **eMethods.** Parallel Analysis Method **eFigure 1.** Internet Platforms and Activities of Concern to Responding Parents **eFigure 2.** Internet Addiction Test (IAT) Scores **eFigure 3.** Respondent Scores for the APQ Domains of Positive Parenting, Inconsistent Discipline, and Poor Monitoring and Supervision **eFigure 4.** Respondent Perception That Internet Use Increases Family Connectedness **eFigure 5.** Reasons Why the Internet Improves Family Connectedness and Mean Impact Score on the Family Due to Internet Use **eFigure 6.** Use of Specific Internet Platforms and Activities by Time

Supplement 2.
Data Sharing Statement
