# Supplementary material for: Parent-Perceived Benefits and Harms Associated With Internet Use by Adolescent Offspring
Source: JAMA Netw Open. 2023 Oct 26;6(10):e2339851. doi: 10.1001/jamanetworkopen.2023.39851 (PMC10603534; doi:10.1001/jamanetworkopen.2023.39851)
Supplement: Supplement 1. — eAppendix. Survey Questionnaire eMethods. Parallel Analysis Method eFigure 1. Internet Platforms and Activities of Concern to Responding Parents eFigure 2. Internet Addiction Test (IAT) Scores eFigure 3. Respondent Scores for the APQ Domains of Positive Parenting, Inconsistent Discipline, and Poor Monitoring and Supervision eFigure 4. Respondent Perception That Internet Use Increases Family Connectedness eFigure 5. Reasons Why the Internet Improves Family Connectedness and Mean Impact Score on the Family Due to Internet Use eFigure 6. Use of Specific Internet Platforms and Activities by Time [file jamanetwopen-e2339851-s001.pdf]

Supplementary Online Content

Kimball HG, Fernandez F, Moskowitz KA, et al. Parent-perceived benefits and harms associated with internet use by adolescent offspring. *JAMA Netw Open*. 2023;6(10):e2339851. doi:10.1001/jamanetworkopen.2023.39851

eAppendix. Survey Questionnaire

eMethods. Parallel Analysis Method

eFigure 1. Internet Platforms and Activities of Concern to Responding Parents

eFigure 2. Internet Addiction Test (IAT) Scores

eFigure 3. Respondent Scores for the APQ Domains of Positive Parenting, Inconsistent Discipline, and Poor Monitoring and Supervision

eFigure 4. Respondent Perception That Internet Use Increases Family Connectedness

eFigure 5. Reasons Why the Internet Improves Family Connectedness and Mean Impact Score on the Family Due to Internet Use

eFigure 6. Use of Specific Internet Platforms and Activities by Time

This supplementary material has been provided by the authors to give readers additional information about their work.

© 2023 Kimball HG et al. *JAMA Network Open*.

Contents

Survey questionnaire 3
About 3
Full Survey Questionnaire 5
Parallel analysis method 33
Parallel Analysis Scree Plot 33
Two-Factor Analysis 33
Makeup of parent mediation and co-parenting factors 34
Figure 1. Internet platforms and activities of concern to responding parents 35
Figure 2. Internet addiction test (IAT) scores a) young people's score as provided by responding parents, b) responding parents of their own internet use 36
a) IAT scores as provided by responding parents on behalf of their young people 36
b) IAT scores provided by responding parents of their own internet use 37
Figure 3. Respondent scores for the APQ domains of Positive Parenting, Inconsistent Discipline and Poor Monitoring and Supervision 38
a) APQ-SF Positive Parenting 38
b) APQ-SF Inconsistent Discipline 38
c) APQ-SF Poor Monitoring and Supervision 39
Figure 4. Respondent perception that Internet use increases family connectedness 40
Figure 5. a) Reasons why the Internet improves family connectedness as cited by respondents; b) mean impact score on the family due to Internet use 41
a) Reasons for improvement 41
b) Mean family impact score 42
Figure 6. Use of specific Internet platforms and activities by time 43

© 2023 Kimball HG et al. *JAMA Network Open*.

eAppendix. Survey Questionnaire

About

Portions of the CRISIS Survey were included to measure demographics, sleep, exercise, media use, and mood circumplex.[1] The 12-item IAT short form was included to assess the problems parents experience in their daily lives due to excessive usage of the internet.[2] Participants completed the IAT for themselves and for their child to gauge excessive child internet use. Questions in the IAT were adapted to replace the word “online”, with “on the internet” for consistency. The IAT has been validated in adult populations and has good reliability with a two-factor solution, Factor 1, Emotional and Cognitive preoccupation with the internet, Factor 2, Loss of Control and Interference with Daily Life. Cronbach's alpha coefficient ranges from $\boldsymbol{\alpha}$ = 0.79–0.88, in addition to having shown good indices for convergent, divergent and incremental validity. The 9-item APQ short form was included to provide information on parent practices and has been validated using a three-factor solution, positive parenting, inconsistent discipline, and poor supervision.[3] Parenting practices were included to explore an understanding of family connectedness as it relates to internet use. The internal consistency reliability was moderate ranging from $\boldsymbol{\alpha}$ =0.59–0.79 in mothers and $\boldsymbol{\alpha}$ =0.63–0.84 in fathers. Five items from the PCS were included to assess parent perception of behavioral control over their children.[4] The PCS is widely used in cross-cultural research and has shown to be reliable and valid as a full measure. Eleven items of 2019 Parent Mediation Items score were included, with the adaptation of ‘smartphone’ to ‘internet use’ for consistency.[5] Four mean indices have been validated, restrictive, active/co-use, monitoring and technical measures. Cronbach’s alpha ranged from $\boldsymbol{\alpha}$ =0.70–0.83. Portions of the EU Kids Online survey were included as a measure of parent-child media use and parent mediation and safety with questions adapted from ‘your child’ to ‘you’.[6] The FACES-IV scale was included to explore family connectedness, with 10-items of the 84-item scale included and internal consistency examined for all six scales (enmeshed, disengaged, balanced cohesion, balanced flexibility and rigid) with Cronbach’s alpha reliable across all.[7] Three items from the 30-item CRPS scale were included to measure parent’s perception of their child’s dependence on the internet.[8] The PROMIS Peer Relationship Scale was adapted to fit the parent respondent cohort with ‘I’ statements rephrased to ‘my child’ to explore parameters of child likeability, peer friendships and social reputation.[9] A series of conversational questions were also included in the survey questionnaire to explore issues that are engaging parents currently, there were presented in understandable language for correlation against other validated survey instruments included in the survey.

References

1. Nikolaidis A, Paksarian D, Alexander L, et al. The Coronavirus Health and Impact Survey (CRISIS) reveals reproducible correlates of pandemic-related mood states across the Atlantic. Sci Rep. Apr 14 2021;11(1):8139. doi:10.1038/s41598-021-87270-3
2. Faraci P, Craparo G, Messina R, Severino S. Internet Addiction Test (IAT): which is the best factorial solution? J Med Internet Res. Oct 9 2013;15(10):e225. doi:10.2196/jmir.2935

© 2023 Kimball HG et al. *JAMA Network Open*.

3. Elgar FJ, Waschbusch DA, Dadds MR, Sigvaldason N. Development and Validation of a Short Form of the Alabama Parenting Questionnaire. Journal of Child and Family Studies. 2007/04/01 2007;16(2):243-259. doi:10.1007/s10826-006-9082-5
4. Rohner RP, Khaleque A. Reliability and Validity of the Parental Control Scale:A Meta-Analysis of Cross-Cultural and Intracultural Studies. Journal of Cross-Cultural Psychology. 2003;34(6):643-649. doi:10.1177/0022022103255650
5. Hefner D, Knop K, Schmitt S, Vorderer P. Rules? Role Model? Relationship? The Impact of Parents on Their Children’s Problematic Mobile Phone Involvement. Media Psychology. 2019/01/02 2019;22(1):82-108. doi:10.1080/15213269.2018.1433544
6. Economics LSo. EU Kids Online Researching European children's online opportunities, risks and safety. Accessed October, 2022. https://www.lse.ac.uk/media-and-communications/research/research-projects/eu-kids-online
7. Olson D. FACES IV and the Circumplex Model: validation study. J Marital Fam Ther. Jan 2011;37(1):64-80. doi:10.1111/j.1752-0606.2009.00175.x
8. Dyer WJ, Kaufman R, Fagan J. Father-child closeness and conflict: Validating measures for nonresident fathers. J Fam Psychol. Dec 2017;31(8):1074-1080. doi:10.1037/fam0000384
9. Devine KA, Willard VW, Hocking MC, et al. PROMIS Peer Relationships Short Form: How Well Does Self-Report Correlate With Data From Peers? Journal of Pediatric Psychology. 2018;43(9):1059-1067. doi:10.1093/jpepsy/jsy038

© 2023 Kimball HG et al. *JAMA Network Open*.

Full Survey Questionnaire

Sample

**QUOTA 1, Total Completes**				
**Cell**	**Description**	**Qualifying Logic**	**Quota/ Target**	**Flexible?**
01	Survey Completes	S95 = 1	1,000	No

**QUOTA 2, Nationally Representative Sample: Race**				
**Cell**	**Description**	**Qualifying Logic**	**Quota/ Target**	**Flexible?**
01	White Non-Hispanic	S25 = 1 AND S25 ≠ 3	573	No
02	Black Non-Hispanic	S25 = 2 AND S25 ≠ 3	97	No
03	Asian Non-Hispanic	S25 = 5,6 AND S25 ≠ 3	69	No
04	Other Non-Hispanic	S25 = 4, 7-9 AND S25 ≠ 3	40	No
05	Hispanic (all races)	S25 = 3	221	No

**QUOTA 3, Nationally Representative Sample: Region**				
**Cell**	**Description**	**Qualifying Logic**	**Quota/ Target**	**Flexible?**
01	Northeast	Based on S05	160	No
02	Midwest	Based on S05	210	No
03	South	Based on S05	390	No
04	West	Based on S05	240	No

**QUOTA 4, Nationally Representative Sample: Race & Gender**				
**Cell**	**Description**	**Qualifying Logic**	**Quota/ Target**	**Flexible?**
01	White Non-Hispanic male	(S25 = 1 AND S25 ≠ 3) AND (S20 = 1)	261	No
02	White Non-Hispanic female	(S25 = 1 AND S25 ≠ 3) AND (S20 = 2)	312	No
03	Black Non-Hispanic male	(S25 = 2 AND S25 ≠ 3) AND (S20 = 1)	44	No
04	Black Non-Hispanic female	(S25 = 2 AND S25 ≠ 3) AND (S20 = 2)	53	No
05	Asian Non-Hispanic male	(S25 = 5,6 AND S25 ≠ 3) AND (S20 = 1)	31	No
06	Asian Non-Hispanic female	(S25 = 5,6 AND S25 ≠ 3) AND (S20 = 2)	38	No
07	Other Non-Hispanic male	(S25 = 4, 7-10 AND S25 ≠ 3) AND (S20 = 1)	18	No
08	Other Non-Hispanic female	(S25 = 4, 7-10 AND S25 ≠ 3) AND (S20 = 2)	22	No
09	Hispanic (all races) male	(S25 = 3) AND (S20 = 1)	101	No
10	Hispanic (all races) female	(S25 = 3) AND (S20 = 2)	120	No

Screener

[SHOW STANDARD INSTRUCTION TEXT ON ALL QUESTIONS; SUPERSEDES Q'AIRE INSTRUCTION TEXT]

© 2023 Kimball HG et al. *JAMA Network Open*.

BASE: ALL RESPONDENTS

S01. In which of the following countries do you reside?

1	United States	
2	United Kingdom	**[TERMINATE]**
3	Canada	**[TERMINATE]**
4	Other (please specify):	**[TERMINATE]**

BASE: ALL RESPONDENTS

S05. In which state is your primary residence located?

[DROP-DOWN LIST OF US STATES + D.C. IN ALPHABETICAL ORDER]

BASE: ALL RESPONDENTS

S10. To confirm, please enter your ZIP code.

______ **[RANGE 0-99999; FORCE 5-DIGIT ENTRY]**

BASE: ALL RESPONDENTS

S15. What is your age?

______ **[RANGE 0-99]**

[TERMINATE IF <18 OR >79]

BASE: ALL RESPONDENTS

S20. What is your gender identity?

1	Male
2	Female
3	Non-binary
4	Transgender male
5	Transgender female
6	Other
7	Prefer not to answer

BASE: ALL RESPONDENTS

S25. Which of these categories best describes you? Select all that apply.

1	White
2	Black or African American
3	Latino / Latina / Latinx or Hispanic
4	American Indian or Alaska Native
5	East Asian or Pacific Islander
6	South or Southeast Asian

© 2023 Kimball HG et al. *JAMA Network Open*.

7	Middle Eastern or North African
8	Caribbean
9	A race, ethnicity, or origin not listed
10	Prefer not to answer

BASE: ALL RESPONDENTS

S30. How many children do you have?

_____ children **[RANGE: 0-15]** **[TERMINATE IF 0]**

BASE: ALL RESPONDENTS

S35. Are you the parent or guardian of any children **age 15 or younger**?

1	Yes
2	No

[TERMINATE]

BASE: PARENTS OF CHILDREN AGE 1-15 (S35 = YES)

S40. How many children for whom you are the parent or guardian are currently living in your household? If there are no applicable children in an age group in your household, please type 0.

[SHOW RUNNING TOTAL]

	**Age Range**	**# in household**
1	8 or younger	
2	9-15	
	**Total**	

[TERMINATE IF 0]

BASE: ALL RESPONDENTS

S95.Please review the information below.

[INSERT PRIVACY LANGUAGE FROM "PRIVACY NOTICE" SECTION AT THE END OF THE QUESTIONNAIRE IN ITS OWN SCROLL BOX. SHOW THE QUESTION TEXT BELOW THE SCROLL BOX.]

By selecting "I Consent" below, you certify that you are eighteen (18) years old or older. You certify that you have read and understand the information above, agree to comply with the requirements included herein and to be a participant in this Market Research.

1	I consent **[CONTINUE]**
2	I do not consent **IF SELECTED SHOW ERROR MESSAGE "Please agree in order to continue" DO NOT ALLOW IF DON'T SELECT "I consent"]**

[SHOW QUALIFICATION MESSAGE ON SEPARATE SCREEN IF QUALIFIED.]

© 2023 Kimball HG et al. *JAMA Network Open*.

Information on Children

BASE: ALL RESPONDENTS

NOTE01. In this survey, we are interested in family behaviors of you and your child(ren). If you have more than one child living at home age 9 to 15, please consider your oldest child in that age group.

[SHOW NOTE01 AND Q105 ON THE SAME PAGE]

BASE: ALL RESPONDENTS

Q105. What is your child's age?

_____ years of age **[RANGE: 9-15]**

BASE: ALL RESPONDENTS

Q110. What is your child's gender identity?

1	Male
2	Female
3	Non-binary
4	Transgender male
5	Transgender female
6	Other
7	Prefer not to answer

BASE: ALL RESPONDENTS

Q115. Which of these categories best describes your child? Select all that apply.

1	White
2	Black or African American
3	Latino / Latina / Latinx or Hispanic
4	American Indian or Alaska Native
5	East Asian or Pacific Islander
6	South or Southeast Asian
7	Middle Eastern or North African
8	Caribbean
9	A race, ethnicity, or origin not listed
10	Prefer not to answer

BASE: ALL RESPONDENTS

Q120. Which best describes the area in which your child lives?

1	Large metropolitan area (population greater than 1.5 million)
2	Metropolitan area (population 500,000-1.5 million)
3	Medium-size urban area (population 200,000-500,000)
4	Small metropolitan area (population 50,000-200,000)

© 2023 Kimball HG et al. *JAMA Network Open*.

5	Large suburban area (population 25,000-50,000)
6	Small suburban area (population 5,000-25,000)
7	Rural area (population less than 5,000)

BASE: ALL RESPONDENTS

Q125. What is your relationship to the child?

1	Mother	
2	Father	
3	Grandparent	
4	Aunt/uncle	
5	Foster parent	
98	Other (please describe):	**[RECORD]**

BASE: ALL RESPONDENTS

Q130. What is the highest level of education **you** completed?

1	Some grade school
2	Some high school
3	High school diploma or GED
4	Some college or 2-year degree
5	4-year college graduate
6	Some school beyond college
7	Graduate or professional degree (e.g., MA, MS, MBA, PhD, EdD, MD, DDS, JD, DVM)

BASE: ALL RESPONDENTS

Q135. Does your child have a second caregiver?

1	Yes
2	No

BASE: CHILD HAS A SECOND CAREGIVER (Q135 = Yes)

Q140. What is their relationship to the child?

1	Mother	
2	Father	
3	Grandparent	
4	Aunt/uncle	
5	Foster parent	
98	Other (please describe):	**[RECORD]**

BASE: CHILD HAS A SECOND CAREGIVER (Q135 = Yes)

Q145. What is the highest level of education your child's other main parent/caregiver completed?

If there is more than one other parent/caregiver, please think about the caregiver who spends the most time with your child.

© 2023 Kimball HG et al. *JAMA Network Open*.

1	Some grade school
2	Some high school
3	High school diploma or GED
4	Some college or 2-year degree
5	4-year college graduate
6	Some school beyond college
7	Graduate or professional degree (e.g., MA, MS, MBA, PhD, EdD, MD, DDS, JD, DVM)

BASE: ALL RESPONDENTS

Q150. What is your **annual household income**?

1	$24,999 or below
2	$25,000 to $49,999
3	$50,000 to $74,999
4	$75,000 to $99,999
5	$100,000 to $124,999
6	$125,000 to $149,999
7	$150,000 to $199,999
8	$200,000 to $249,999
9	$250,000 to $299,999
10	$300,000 or more
11	Prefer not to answer

BASE: ALL RESPONDENTS

Q155. How many people are living or staying at your current address? Include yourself and any other adults or children who are currently living or staying at this address.

_____ **[RANGE: 2-99]**

BASE: ALL RESPONDENTS

Q160. Has a health or education professional ever said that <u>your child had</u> any of the following?

Please select all that apply.

[RANDOMIZE]

1	Depression	
2	Anxiety	
3	Problems with alcohol or drugs	
4	Post-traumatic Stress Disorder (PTSD)	
5	Attention Deficit Hyperactivity Disorder (ADHD)	
6	Intellectual disability	
7	Disruptive Behaviors	
8	Autism Spectrum Disorder	
9	Learning Disorder	
13	Schizophrenia or Psychosis	
10	Other (please describe):	**[ANCHOR; RECORD]**
11	None of the above	**[ANCHOR; MUTUALLY EXCLUSIVE]**

© 2023 Kimball HG et al. *JAMA Network Open*.

12	Prefer not to answer	**[ANCHOR; MUTUALLY EXCLUSIVE]**

BASE: CHILD HAS A HEALTH CONDITION (Q160 ≠ "None of the above" OR "Prefer not to answer")

Q165. Does this condition / these conditions interfere with your child's daily life?

1	Yes
2	No

BASE: ALL RESPONDENTS

Q170. Has your child received mental health care in the past 12 months?

1	Yes
2	No

BASE: ALL RESPONDENTS

Q175. Has a health or education professional ever said that someone in your family, other than your child, has any of the following?

Please select all that apply.

[RANDOMIZE]

1	Depression	
2	Anxiety	
3	Problems with alcohol or drugs	
4	Post-traumatic Stress Disorder (PTSD)	
5	Attention Deficit Hyperactivity Disorder (ADHD)	
6	Intellectual disability	
7	Disruptive Behaviors	
8	Autism Spectrum Disorder	
9	Learning Disorder	
10	Schizophrenia or Psychosis	
11	Other (please describe):	**[ANCHOR; RECORD]**
12	None of the above	**[ANCHOR; MUTUALLY EXCLUSIVE]**
13	Prefer not to answer	**[ANCHOR; MUTUALLY EXCLUSIVE]**

BASE: ALL RESPONDENTS

Q180. Has a health or education professional ever said that your child had any of the following health conditions?

Please select all that apply.

[RANDOMIZE]

1	Allergies

© 2023 Kimball HG et al. *JAMA Network Open*.

2	Asthma or other lung problems	
3	Heart problems or hypertension	
4	Kidney problems	
5	Immune disorder	
6	Diabetes or high blood sugar	
7	Cancer	
8	Arthritis	
9	Frequent or very bad headaches	
10	Epilepsy or seizures	
11	Serious stomach or bowel problems	
12	Serious acne or skin problems	
13	None of the above	**[ANCHOR; MUTUALLY EXCLUSIVE]**
14	Prefer not to answer	**[ANCHOR; MUTUALLY EXCLUSIVE]**

BASE: ALL RESPONDENTS

Q185. Has a health or education professional ever said that someone else other than your child in your family has any of the following health conditions?

Please select all that apply.

[RANDOMIZE]

1	Allergies	
2	Asthma or other lung problems	
3	Heart problems or hypertension	
4	Kidney problems	
5	Immune disorder	
6	Diabetes or high blood sugar	
7	Cancer	
8	Arthritis	
9	Frequent or very bad headaches	
10	Epilepsy or seizures	
11	Serious stomach or bowel problems	
12	Serious acne or skin problems	
13	None of the above	**[ANCHOR; MUTUALLY EXCLUSIVE]**
14	Prefer not to answer	**[ANCHOR; MUTUALLY EXCLUSIVE]**

Internet Usage

BASE: ALL RESPONDENTS

NOTE02. We would like to ask you questions about your and your child's internet usage. In this survey, "internet" means:

© 2023 Kimball HG et al. *JAMA Network Open*.

- **Connected computers and web browsing**
- **Email and other messaging**
- **Mobile phones (whether used for phone calls, messaging, social media, or accessing streaming media or gaming)**
- **Connected devices like portable game consoles**
- **Digital media**
- **Streaming video**
- **Internet TV**
- **Gaming consoles connected to online communities**

[SHOW NOTE02 AND Q210 ON THE SAME PAGE]

BASE: ALL RESPONDENTS
Q210. Does your child have internet access at home?

1	Yes
2	No

BASE: ALL RESPONDENTS
Q215. Does your child have a personal internet enabled device? (e.g. tablet, cell phone)?

1	Yes
2	No

BASE: RESPONDENTS' CHILD HAS INTERNET ACCESS AT HOME (Q210 = Yes)
Q220. How reliable is the internet connection your child uses at home?

Very unreliable	Somewhat unreliable	Somewhat reliable	Very reliable
4	3	2	1
O	O	O	O

BASE: ALL RESPONDENTS
Q225. In the last two weeks, on average, how many hours **per day** did your child spend…

	[RANDOMIZE]	None	Under 1 hour	1-3 hours	4-6 hours	More than 6 hours	I don't know
	Data Code	1	2	3	4	5	6
1	Using the internet for general purposes (e.g. web browsing, shopping, news, podcasts, music)	O	O	O	O	O	O
2	Watching streaming video (e.g. YouTube,	O	O	O	O	O	O

© 2023 Kimball HG et al. *JAMA Network Open*.

	Twitch, Netflix, Digital TV)						
3	Using content sharing apps (e.g. TikTok, Instagram, Triller, VSCO)	0	0	0	0	0	0
4	Using social networking apps (e.g. Facebook, Pinterest, Tumblr, Twitter)	0	0	0	0	0	0
5	Playing video games (e.g. Among Us, Call of Duty, Fortnite, GTA, Minecraft, Nintendo Switch, Roblox)	0	0	0	0	0	0
6	Using messaging or forums apps (e.g. Reddit, WhatsApp, Snapchat, Discord, Telegram, Messenger, Kik, Marco Polo, Hangouts)	0	0	0	0	0	0
7	In immersive online environments such as virtual and augmented reality (e.g. Houseparty, Gathertown, Second Life, Nowhere)	0	0	0	0	0	0

BASE: HAVE CHILDREN WHO USED INTERNET IN PAST TWO WEEKS (Q225 IS NOT "NONE" FOR EVERY OPTION)

Q230. What devices did your child use for the following?

Please select all that apply.

	COLUMNS: SHOW COLUMN IF Q225 = 2,3,4,5 FOR THAT ROW/ACTIVITY [ROWS: RANDOMIZE]	Using the internet for general purposes	Watching streaming video	Using content sharing apps	Using social networking apps	Playing video games	Using messaging or forums apps	In immersive environments such as virtual and augmented reality
	Data Code	1	2	3	4	5	6	7
1	Parent's mobile phone/smart phone	0	0	0	0	0	0	0

© 2023 Kimball HG et al. *JAMA Network Open*.

2	Child's personal mobile phone/smart phone	0	0	0	0	0	0	0
3	TV/smart TV	0	0	0	0	0	0	0
4	Parent's tablet	0	0	0	0	0	0	0
5	Child's personal tablet							
6	Desktop computer, laptop, or notebook computer	0	0	0	0	0	0	0
7	Gaming console	0	0	0	0	0	0	0
8	A Virtual Reality Device (e.g., VR Headset)	0	0	0	0	0	0	0
9	Voice activated device (e.g., Alexa, Siri)	0	0	0	0	0	0	0
10	Other (please describe):	0	0	0	0	0	0	0

BASE: ALL RESPONDENTS

Q235. How often do the following occur with <u>your child</u>? How often…

	**[RANDOMIZE; REPEAT HEADER EVERY 4 STATEMENTS]**	**Rarely**	**Occasionally**	**Frequently**	**Often**	**Always**	**Does not apply**
	Data Code	1	2	3	4	5	99
1	Do you find that your child stays on the internet longer than they intended?	0	0	0	0	0	0
2	Do you find your child saying "just a few more minutes" when on the internet?	0	0	0	0	0	0
3	Does your child neglect household chores to spend more time on the internet?	0	0	0	0	0	0
4	Does your child try to cut down the amount of time they spend on the internet and fail?	0	0	0	0	0	0
5	Do your child's grades or schoolwork suffer because of	0	0	0	0	0	0

© 2023 Kimball HG et al. *JAMA Network Open*.

	the amount of time they spend on the internet?						
6	Does your child lose sleep due to being online late at night?	O	O	O	O	O	O
7	Does your child choose to spend more time on the internet over going out with others?	O	O	O	O	O	O
8	Does your child try to hide how long they've been on the internet?	O	O	O	O	O	O
9	Does your child snap, yell, or act annoyed if someone bothers them while they are on the internet?	O	O	O	O	O	O
10	Does your child feel depressed, moody, or nervous when they are off-line, which goes away once they are back on the internet?	O	O	O	O	O	O
11	Does your child feel preoccupied with the Internet when off-line, or fantasize about being on the internet?	O	O	O	O	O	O
12	Does your child become defensive or secretive when anyone asks them what they do on the internet?	O	O	O	O	O	O

BASE: ALL RESPONDENTS

Q240. Now think about your personal internet use. In the last two weeks, on average, how many hours **per day** did you spend...

	[RANDOMIZE]	None	Under 1 hour	1-3 hours	4-6 hours	More than 6 hours
	Data Code	1	2	3	4	5
1	Using the internet for general purposes (e.g. web browsing, shopping, news, podcasts, music)	O	O	O	O	O
2	Watching streaming video (e.g. YouTube, Twitch, Netflix, Digital TV)	O	O	O	O	O
3	Using content sharing apps (e.g. TikTok, Instagram, Triller, VSCO)	O	O	O	O	O
4	Using social networking apps (e.g. Facebook, Pinterest, Tumblr, Twitter)	O	O	O	O	O

© 2023 Kimball HG et al. *JAMA Network Open*.

5	Playing video games (e.g. Among Us, Call of Duty, Fortnite, GTA, Minecraft, Nintendo Switch, Roblox)	O	O	O	O	O
6	Using messaging or forums apps (e.g. Reddit, WhatsApp, Snapchat, Discord, Telegram, Messenger, Kik, Marco Polo, Hangouts)	O	O	O	O	O
7	In immersive online environments such as virtual and augmented reality (e.g. Houseparty, Gathertown, Second Life, Nowhere)	O	O	O	O	O

BASE: USED INTERNET IN PAST TWO WEEKS (Q240 IS NOT "NONE" FOR EVERY OPTION)

Q245. What devices did you use for the following?

Please select all that apply.

	**COLUMNS: SHOW COLUMN IF Q240 = 2,3,4,5 FOR THAT ROW/ACTIVITY [ROWS: RANDOMIZE]**	**Using the internet for general purposes**	**Watching streaming video**	**Using content sharing apps**	**Using social networking apps**	**Playing video games**	**Using messaging or forums apps**	**In immersive environments such as virtual and augmented reality**
	Data Code	1	2	3	4	5	6	7
1	Mobile phone/smart phone	O	O	O	O	O	O	O
2	TV/smart TV	O	O	O	O	O	O	O
3	Tablet	O	O	O	O	O	O	O
4	Desktop computer, laptop, or notebook computer	O	O	O	O	O	O	O
5	Gaming console	O	O	O	O	O	O	O
6	A Virtual Reality Device (e.g., VR Headset)	O	O	O	O	O	O	O
7	Voice activated	O	O	O	O	O	O	O

© 2023 Kimball HG et al. *JAMA Network Open*.

	device (e.g., Alexa, Siri)							
8	Other (please describe):	O	O	O	O	O	O	O

BASE: ALL RESPONDENTS

Q250. How often do the following occur? How often do you...

	[RANDOMIZE; REPEAT HEADER EVERY 4 STATEMENTS]	Rarely	Occasionally	Frequently	Often	Always	Does not apply
	Data Code	1	2	3	4	5	6
1	Find that you stay on the internet than you intended	O	O	O	O	O	O
2	Find yourself saying "just a few more minutes" when on the internet	O	O	O	O	O	O
3	Neglect household chores to spend more time on-line	O	O	O	O	O	O
4	Try to cut down the amount of time you spend on the internet and fail	O	O	O	O	O	O
5	Find your work suffers because of the amount of time you spend on the internet	O	O	O	O	O	O
6	Lose sleep due to being online late at night	O	O	O	O	O	O
7	Choose to spend more time on the internet over going out with others	O	O	O	O	O	O
8	Try to hide how long you've been on the internet	O	O	O	O	O	O
9	Snap, yell or act annoyed if someone bothers you while you are on the internet	O	O	O	O	O	O
10	Feel depressed, moody, or nervous when you are off-line, which goes away once you are back on the internet	O	O	O	O	O	O
11	Feel preoccupied with the Internet when off-line, or fantasize about being on the internet	O	O	O	O	O	O
12	Become defensive or secretive when anyone asks you what you do on the internet	O	O	O	O	O	O

© 2023 Kimball HG et al. *JAMA Network Open*.

Family Dynamics

BASE: ALL RESPONDENTS
NOTE03. Now, we'd like to show you a number of statements about your family.

[SHOW NOTE03 AND Q305 ON THE SAME PAGE]

BASE: ALL RESPONDENTS
Q305. Please rate each item as to how often it typically occurs in your home.

	[RANDOMIZE; REPEAT HEADER EVERY 3 STATEMENTS]	Never	Almost Never	Someti mes	Often	Alway s
	Data Code	1	2	3	4	5
1	You let your child know when he/she is doing a good job with something	O	O	O	O	O
2	You threaten to punish your child and then do not actually punish him/her	O	O	O	O	O
3	Your child fails to leave a note or to let you where he/she is going	O	O	O	O	O
4	Your child talks you out of being punished after he/she has done something wrong	O	O	O	O	O
5	Your child stays out in the evening after the time he/she is supposed to be home	O	O	O	O	O
6	You compliment your child after he/she has done something well	O	O	O	O	O
7	You praise your child if he/she behaves well	O	O	O	O	O
8	Your child is out with friends you don't know	O	O	O	O	O
9	You let your child out of a punishment early (like lift restrictions earlier than you originally said)	O	O	O	O	O

BASE: ALL RESPONDENTS
Q310. How often are the following statements true?

	[RANDOMIZE]	Almost never true	Rarely true	Sometime s true	Almost always true
	Data Code	4	3	2	1
1	I see to it that my child knows exactly what (s)he may or may not do	O	O	O	O

© 2023 Kimball HG et al. *JAMA Network Open*.

2	I always tell my child how (s)he should behave	O	O	O	O
3	I insist my child does exactly as (s)he is told	O	O	O	O
4	I let my child do anything (s)he would like to do	O	O	O	O
5	I want to control whatever my child does	O	O	O	O

BASE: ALL RESPONDENTS

Q315. To what extent do you agree with the statements below?

	[RANDOMIZE; REPEAT HEADER EVERY 4 STATEMENTS]	Strongly disagree	Disagree	Neutral / Unsure / neither agree nor disagree	Agree	Strongly agree
	Data Code	1	2	3	4	5
1	I often explain things regarding the internet to my child	O	O	O	O	O
2	I often talk with my child about what he or she is doing on the internet	O	O	O	O	O
3	My child and I often use the internet together because it is fun and we share the same interests	O	O	O	O	O
4	I often let my child show me what he/she is doing on the internet	O	O	O	O	O
5	I often prohibit specific activities concerning my child on the internet	O	O	O	O	O
6	I often ask my child to stop when on the internet, because it is too late, or they have already been on the internet for a long time	O	O	O	O	O
7	I define time frames for my child's internet use or restrict the duration of usage	O	O	O	O	O
8	I often monitor what my child does on the internet sometimes even after the usage	O	O	O	O	O
9	I usually track the persons my child was in contact with on the internet	O	O	O	O	O

© 2023 Kimball HG et al. *JAMA Network Open*.

10	I apply technical measures in order to block or filter specific contents on my child's internet connected devices	O	O	O	O	O
11	I apply technical measures in order to check what my child has done on the internet	O	O	O	O	O

BASE: ALL RESPONDENTS

Q320. Regarding internet usage, please rate each of the following statements in terms of how often each has occurred in your home.

	[RANDOMIZE]	Never	Hardly ever	Sometimes	Often	Very often	I don't know
	Data Code	1	2	3	4	5	6
1	You talk to your child about what to do if something online bothers or upsets them when your child uses the internet	O	O	O	O	O	O
2	Your child tells you about things that bother or upset them on the internet	O	O	O	O	O	O
3	Your child starts a discussion with you about what they do on the internet	O	O	O	O	O	O
4	Your child asks for your advice on how they should act on the internet	O	O	O	O	O	O

BASE: ALL RESPONDENTS

Q325. How would you describe the impact of internet on each of the following?

	[RANDOMIZE; REPEAT HEADER EVERY 5 STATEMENTS]	Very harmful	Somewhat harmful	No impact	Somewhat beneficial	Very beneficial
	Data Code	1	2	3	4	5
1	The degree of closeness between family members	O	O	O	O	O

© 2023 Kimball HG et al. *JAMA Network Open*.

2	Your family's ability to cope with stress	O	O	O	O	O
3	Your family's ability to be flexible	O	O	O	O	O
4	Your family's ability to share positive experiences	O	O	O	O	O
5	The quality of communication between family members	O	O	O	O	O
6	Your family's ability to resolve conflicts	O	O	O	O	O
7	The amount of time you spend together as a family	O	O	O	O	O
8	The way problems are discussed	O	O	O	O	O
9	The fairness of criticism in your family	O	O	O	O	O
10	Family members' concern for each other	O	O	O	O	O

BASE: ALL RESPONDENTS

Q330. To what extent do you believe the following statements apply to you?

	[RANDOMIZE]	Definitely does not apply to me	Not really	Neutral / not sure	Applies somewhat	Definitely applies
	Data Code	1	2	3	4	5
1	My child appears hurt or embarrassed when I correct him/her	O	O	O	O	O
2	My child reacts strongly to separation from me	O	O	O	O	O
3	My child is overly dependent on me	O	O	O	O	O

BASE: ALL RESPONDENTS

Q335. The following questions ask about a coparent. This is someone you share primary parenting responsibilities with. Please indicate who you consider your coparent?

1	Your child's biological parent, living together
2	Your child's biological parent, living apart
3	Your child's stepparent
4	Your child's foster parent
5	Your child's grandparent

© 2023 Kimball HG et al. *JAMA Network Open*.

6	Your partner/significant Other
7	I do not share parenting responsibilities with anyone
8	Other (please describe): **[RECORD]**

BASE: RESPONDENTS WITH A COPARENT (Q335 ≠ "I do not share parenting responsibilities with anyone")

Q340. When it comes to monitoring and supporting healthy internet use in our household...

	[RANDOMIZE]	Not true of us 0	1	A little bit true of us 2	3	Some what true of us 4	5	Very true of us 6
	Data Code	0	1	2	3	4	5	6
1	I believe my coparent is a good parent	0	0	0	0	0	0	0
2	My coparent pays a great deal of attention to our child	0	0	0	0	0	0	0
3	My coparent likes to play with our child and then leave the dirty work to me	0	0	0	0	0	0	0
4	My coparent and I have the same goals for our child	0	0	0	0	0	0	0
5	My coparent does not carry his or her fair share of the parenting work when it comes to internet use	0	0	0	0	0	0	0
6	My coparent undermines my parenting when it comes to internet use	0	0	0	0	0	0	0
7	My coparent and I argue in front of the child	0	0	0	0	0	0	0

BASE: ALL RESPONDENTS

Q345. Over the past two weeks...

	[RANDOMIZE; REPEAT HEADER EVERY 4 STATEMENTS]]	Never	Almost Never	Someti mes	Often	Alway s	I don't know
	Data Code	1	2	3	4	5	6
1	My child felt accepted by other kids their age	0	0	0	0	0	0
2	My child was able to count on their friends	0	0	0	0	0	0

© 2023 Kimball HG et al. *JAMA Network Open*.

3	My child was able to talk about everything with their friends	O	O	O	O	O	O
4	My child was good at making friends	O	O	O	O	O	O
5	My child and their friends helped each other out	O	O	O	O	O	O
6	Other kids wanted my child to be their friend	O	O	O	O	O	O
7	Other kids wanted to be with my child	O	O	O	O	O	O
8	Other kids wanted to talk to my child	O	O	O	O	O	O
9	My child is picked on or bullied by other children	O	O	O	O	O	O

Sleep, Mood, and Outdoor Activities

[INTRO; SHOW ON SEPARATE SCREEN]
For the next set of questions, please think about the last two weeks.

BASE: ALL RESPONDENTS
Q405. Over the past two weeks, on average, how much sleep did your child get…

		Fewer than 6 hours	6-8 hours	8-10 hours	More than 10 hours
	Data Code	1	2	3	4
1	On weeknights	O	O	O	O
2	On weekends	O	O	O	O

BASE: ALL RESPONDENTS
Q410. Over the past two weeks, how often did your child have trouble sleeping?

1	Not at all during the past 2 weeks
2	Less than once a week
3	Once or twice a week
4	Three or more times a week
5	I don't know

BASE: ALL RESPONDENTS
Q415. Over the past two weeks, how was your child's sleep quality overall?

© 2023 Kimball HG et al. *JAMA Network Open*.

Very bad	Fairly bad	Fairly good	Very good
4	3	2	1
O	O	O	O

[SHOW Q420 AND Q425 ON THE SAME PAGE]

BASE: ALL RESPONDENTS

Q420. Over the past two weeks, how many days per week did your child **exercise** (increased heart rate, heavier breathing) for at least 30 minutes?

1	None
2	1-2 days
3	3-4 days
4	5-6 days
5	Everyday
6	I don't know

BASE: ALL RESPONDENTS

Q425. Over the past two weeks, how many days per week did your child spend time **outdoors** for at least 30 minutes?

1	None
2	1-2 days
3	3-4 days
4	5-6 days
5	Everyday
6	I don't know

[RANDOMIZE ORDER OF Q430-Q475; SHOW THREE QUESTIONS PER PAGE; RANDOMIZE SCALE ORDER "GOOD TO BAD" AND "BAD TO GOOD" FOR RESPONDENTS BUT DO NOT FULLY RANDOMIZE SCALES]

BASE: ALL RESPONDENTS

Q430. During the past two weeks, how **worried** was your child generally?

Extremely Worried	Very Worried	Moderately Worried	Slightly Worried	Not worried at all
5	4	3	2	1
O	O	O	O	O

BASE: ALL RESPONDENTS

Q435. During the past two weeks, how **happy versus sad** was your child?

Very sad / depressed / moody	Moderately sad / depressed / unhappy	Neutral	Moderately happy / cheerful	Very happy / cheerful
1	2	3	4	5

© 2023 Kimball HG et al. *JAMA Network Open*.

0	0	0	0	0

BASE: ALL RESPONDENTS

Q440. During the past two weeks, how much had your child been able to **enjoy his/her usual activities**?

Not at all	Slightly	Moderately	Very Much	A lot
1	2	3	4	5
0	0	0	0	0

BASE: ALL RESPONDENTS

Q445. During the past two weeks, how **relaxed versus anxious** was your child?

Very nervous / anxious	Moderately nervous / anxious	Neutral	Moderately calm / relaxed	Very relaxed / calm
1	2	3	4	5
0	0	0	0	0

BASE: ALL RESPONDENTS

Q450. During the past two weeks, how **fidgety or restless** was your child?

Extremely fidgety / restless	Very fidgety / restless	Moderately fidgety / restless	Slightly fidgeting / restless	Not fidgety / restless at all
5	4	3	2	1
0	0	0	0	0

BASE: ALL RESPONDENTS

Q455. During the past two weeks, how **fatigued or tired** was your child?

Extremely fatigued or tired	Very fatigued or tired	Moderately fatigued or tiered	Slightly fatigued or tired	Not fatigued or tired at all
5	4	3	2	1
0	0	0	0	0

BASE: ALL RESPONDENTS

Q460. During the past two weeks, how well was your child able to **concentrate or focus**?

Very unfocused / distracted	Moderately unfocused / distracted	Neutral	Moderately focused / attentive	Very focused / attentive
1	2	3	4	5
0	0	0	0	0

BASE: ALL RESPONDENTS

© 2023 Kimball HG et al. *JAMA Network Open*.

Q465. During the past two weeks, how **irritable or easily angered** was your child?

Extremely irritable or easily angered	Very irritable or easily angered	Moderately irritable or easily angered	Slightly irritable or easily angered	Not irritable or easily angered at all
5	4	3	2	1
O	O	O	O	O

BASE: ALL RESPONDENTS

Q470. During the past two weeks, how **lonely** was your child?

Extremely lonely	Very lonely	Moderately lonely	Slightly lonely	Not lonely at all
5	4	3	2	1
O	O	O	O	O

BASE: ALL RESPONDENTS

Q475. During the past two weeks, to what extent did your child express negative thoughts or things that made them feel bad?

A lot of the time	Often	Occasionally	Rarely	Not at all
5	4	3	2	1
O	O	O	O	O

Internet Responsibility

BASE: ALL RESPONDENTS

Q505. The following are general questions about your feelings about your child's internet use. To what extent do you agree with the statements below?

	[RANDOMIZE; REPEAT HEADER EVERY 5 STATEMENTS]	Strongly disagree	Disagree	Neutral / Unsure/ neither agree nor disagree	Agree	Strongly agree
	Data Code	1	2	3	4	5
1	I believe my child is able to use the internet responsibly	O	O	O	O	O
2	I believe the internet is good for my child's social development	O	O	O	O	O

3	I feel comfortable talking about my child's use of the internet with my child	0	0	0	0	0
4	I feel comfortable talking about mental health with my child	0	0	0	0	0
5	I am concerned about how the internet will impact my child's social and emotional development	0	0	0	0	0
6	I am concerned about how the internet will impact my child's cognitive or academic development	0	0	0	0	0
7	I am concerned about how the internet will impact my child's physical health					
8	I am concerned about the content / information that is available online for my child	0	0	0	0	0
9	I am concerned about the impact of online bullying or criticism on my child	0	0	0	0	0
10	I believe the internet increases the connectedness of my child with our nuclear family	0	0	0	0	0
11	I believe the internet increases the connectedness of my child with our extended family	0	0	0	0	0
12	I am confident in my ability to know how much screen time is appropriate for my child	0	0	0	0	0
13	I am often distracted by the internet while spending time with my children	0	0	0	0	0
14	The adults in our household have a consistent strategy regarding how to handle internet use in the family	0	0	0	0	0
15	I feel helpless on how to limit internet technologies for my children	0	0	0	0	0
16	I feel confident I know alternatives to internet use and screen time that I can provide to my child	0	0	0	0	0
17	I use the internet to distract, calm or pacify my child	0	0	0	0	0
18	I believe my attitude toward my child's screen time and internet use has become more permissive during the pandemic	0	0	0	0	0
19	My child talks about having a negative relationship with the internet/internet usage	0	0	0	0	0

© 2023 Kimball HG et al. *JAMA Network Open*.

20	My child seems to feel worse about themselves after using the internet	O	O	O	O	O
21	I try to be a role model about healthy internet usage for my child	O	O	O	O	O
22	I make an effort to use the internet together with my child as a parenting strategy	O	O	O	O	O

BASE: RESPONDENTS CONFIDENT THEY KNOW HOW MUCH SCREEN TIME IS APPROPRIATE (Q505_12 = 4,5)

Q510. You stated that you are confident in your ability to know how much screen time is appropriate for your child. Please indicate how much screen time you feel is appropriate for your child per day.

[SELECT ONE]

1	Under 1 hour
2	1-3 hours
3	4-6 hours
4	More than 6 hours

BASE: RESPONDENTS CONFIDENT THEY KNOW ALTERNATIVE TO INTERNET USE & SCREEN TIME (Q505_16 = 4,5)

Q515. You stated that you feel confident you know alternatives to internet use and screen time for your child. Please indicate what alternatives you provide your child.

[LARGE OPEN TEXT BOX]

BASE: ALL RESPONDENTS

Q520. To what extent do you agree with the statements below?

	[RANDOMIZE]	Strongly disagree	Disagree	Neutral / Unsure/ neither agree nor disagree	Agree	Strongly agree
	Data Code	1	2	3	4	5
1	Children and adolescents can be addicted to internet technologies much the same way they can be addicted to substances.	O	O	O	O	O

© 2023 Kimball HG et al. *JAMA Network Open*.

2	Technology companies should play a more active role in regulation of the internet for use by children and adolescent	O	O	O	O	O
3	The government should play a more active role in regulation of the internet for use by children and adolescents	O	O	O	O	O
4	My child receives satisfactory instruction on safe internet use in school	O	O	O	O	O

BASE: ALL RESPONDENTS

Q525. Are you worried about internet addiction and substance addiction for your child?

[RANDOMIZE OPTIONS 1 AND 2]

1	Internet addiction
2	Substance addiction
3	Equally worried about both
4	Neither

BASE: ALL RESPONDENTS

Q530. Which of the following is your most trusted source of information about children's internet use for your family?

[RANDOMIZE]

1	School
2	Pediatrician / mental health provider
3	Scientists
4	Community / religious group
5	None of the above **[ANCHOR]**

BASE: ALL RESPONDENTS

Q535. Which internet technology are you most worried about for your child?

[SELECT ONE; RANDOMIZE]

1	General purposes (e.g., web browsing, shopping, news, podcasts, music)
2	Streaming video
5	Content sharing apps
3	Social networking apps
4	Video games
6	Messaging or forum apps
7	Immersive online environments

© 2023 Kimball HG et al. *JAMA Network Open*.

8	None, I am not concerned about my child's use of internet technologies **[ANCHOR]**

BASE: CONCERNED ABOUT TECHNOLOGY (Q535 IS NOT "NONE" (8))

Q575. Please describe why you are most worried about this technology~~platform~~.

[OPEN-ENDED]

[SHOW Q575 ON SAME PAGE WITH Q535]

Open-ended Feedback

BASE: ALL RESPONDENTS

Q605. How have your expectations of your child's screen use changed during the pandemic?

[OPEN-ENDED]

BASE: ALL RESPONDENTS

Q607. The **metaverse** is increasingly believed to be the next iteration of the Internet. The metaverse is composed of *1) Virtual reality: digital representations of physical spaces, and 2) Augmented reality: digital overlays on top of physical spaces*.

How prepared do you feel for parenting in the age of the metaverse?

[SELECT ONE; RANDOMIZE SCALE ORDER "GOOD TO BAD" AND "BAD TO GOOD" FOR RESPONDENTS BUT DO NOT FULLY RANDOMIZE SCALES]

1	Not prepared at all
2	Slightly prepared
3	Moderately prepared
4	Very prepared
5	Extremely prepared
6	I don't know

BASE: ALL RESPONDENTS

Q610. Please explain why you selected this answer below.

[OPEN-ENDED]

[SHOW Q607 & Q610 ON THE SAME PAGE]

BASE: ALL RESPONDENTS

© 2023 Kimball HG et al. *JAMA Network Open*.

Q615. How do you feel your own use of the internet impacts your family dynamics?

[OPEN-ENDED]

Demographics

[SHOW ON SAME PAGE AS Q905]: These last couple questions are for classification purposes only.

BASE: ALL RESPONDENTS

Q905. What is your marital status?

1	Married
2	Domestic Partnership
3	Separated
4	Divorced
5	Never Married
6	Widowed

BASE: ALL RESPONDENTS

Q910. Which of the following best describes your primary form of health insurance?

1	Commercial Insurance (e.g. Anthem, United Healthcare, etc.)
2	Medicaid or similar state-sponsored health insurance
3	Veterans/Military
4	Medicare without supplemental coverage
5	Medicare with supplemental coverage
6	Medicare Advantage
7	Other type of coverage
8	No coverage / cash paying
99	Not sure **[EXCLUSIVE]**

© 2023 Kimball HG et al. *JAMA Network Open*.

eMethods. Parallel Analysis Method

Parallel Analysis Scree Plot

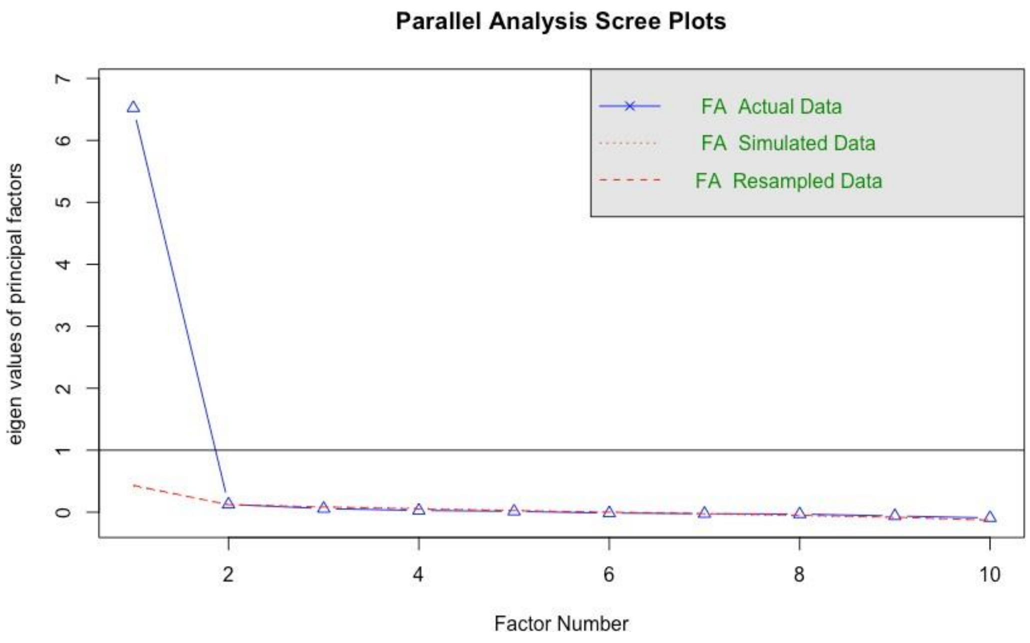

Two-Factor Analysis

Items	Standardized loadings		h2
	Factor 1	Factor 2	
1. Degree of closeness within family	**0.84**	-0.15	0.71
2. Family's ability to cope with stress	**0.78**	0.09	0.68
3. Family's ability to be felxible	**0.81**	-0.01	0.66
4. Family's ability to share positive experiences	**0.79**	-0.07	0.62
5. Quality of communicatioin between family	**0.83**	-0.15	0.69
6. Family's ability to resolve conflicts	**0.84**	0.1	0.72
7. The way problems are discussed	**0.8**	-0.11	0.65
8. The amount of time spent together as a family	**0.8**	0.2	0.69
9. Fairness of criticism in family	**0.77**	0.18	0.64
10. Family members' concern for each other	**0.82**	-0.01	0.67
Eigenvalues	**6.54**	0.16	
Percentage explained variance	**0.98**	0.2	

© 2023 Kimball HG et al. *JAMA Network Open*.

Makeup of parent mediation and co-parenting factors

The first parent mediation factors (positive parent mediation) included explaining things regarding the internet, going on the internet together, and sharing interests on the internet. The second factor for parent mediation (restrictive parent mediation) included prohibiting activities on the internet, asking their offspring to stop, and defining time frames for internet use. The third parent mediation factor (technical mediation) included applying technical measures to check, monitor or block their offspring's internet usage.

The first co-parenting factor (Positive Perception of co-parent) included items that had positive perception of their co-parent such as sharing the same goals as co-parents and believing that the co-parent is a good parent. On the other hand, the second co-parenting factor (Negative perceptions of co-parent) included items that had negative perceptions of their co-parent such as: co-parent not carrying their share of parenting work and co-parents arguing and undermining each other.

© 2023 Kimball HG et al. *JAMA Network Open*.

eFigure 1. Internet Platforms and Activities of Concern to Responding Parents

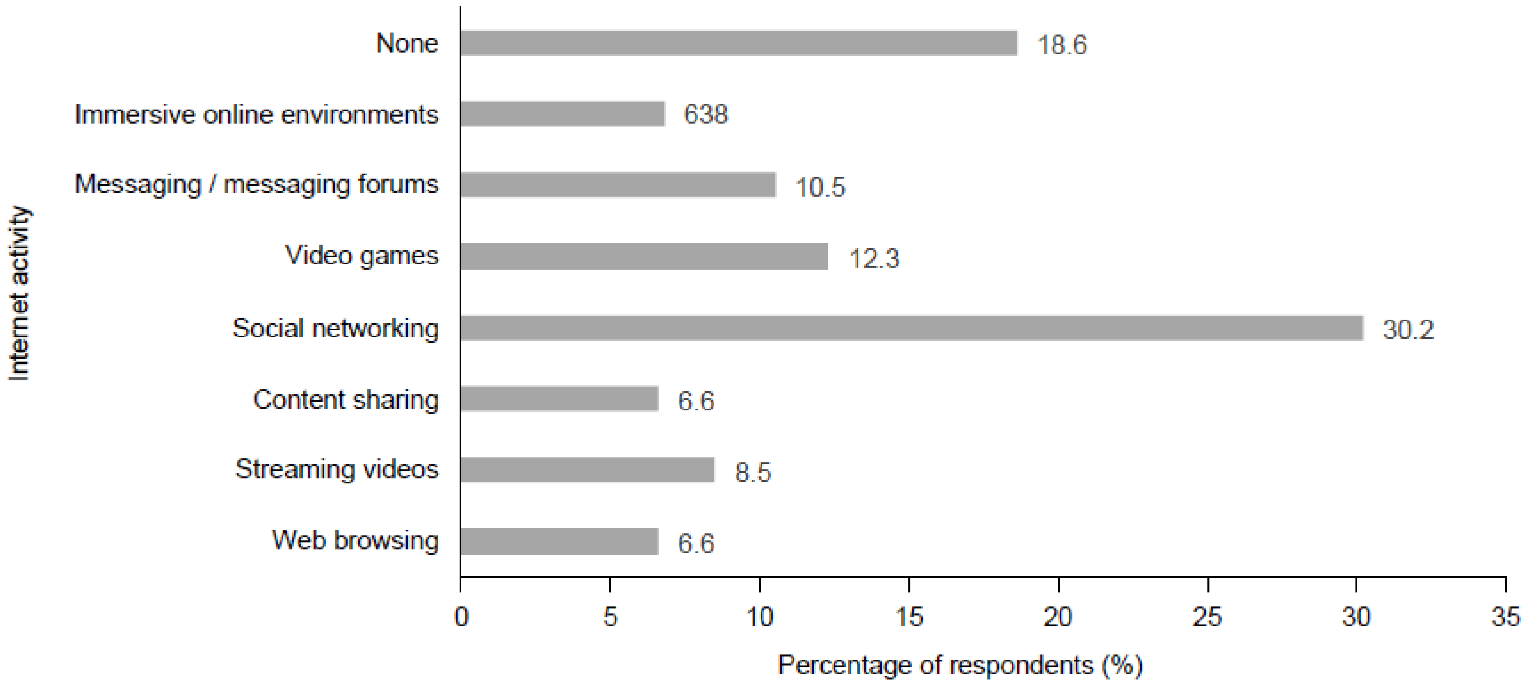

© 2023 Kimball HG et al. *JAMA Network Open*.

eFigure 2. Internet Addiction Test (IAT) Scores

a) IAT scores as provided by responding parents on behalf of their young people

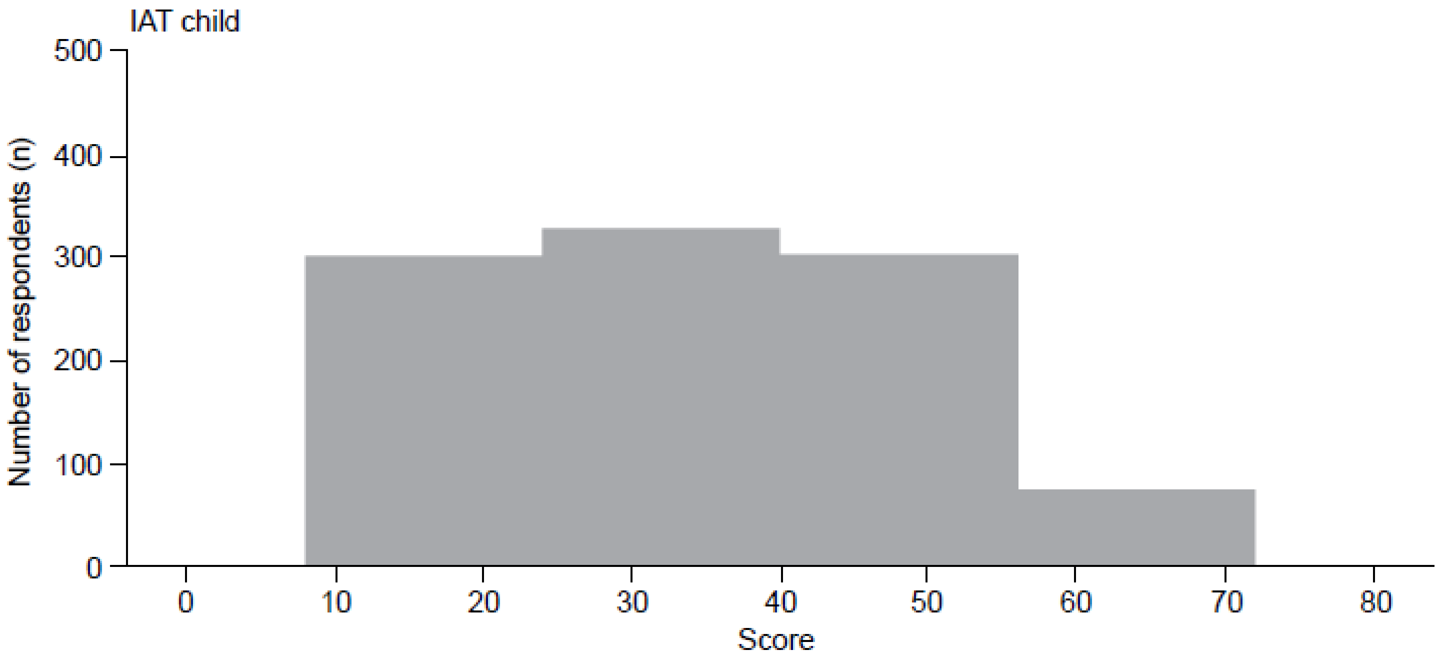

© 2023 Kimball HG et al. *JAMA Network Open*.

b) IAT scores provided by responding parents of their own internet use

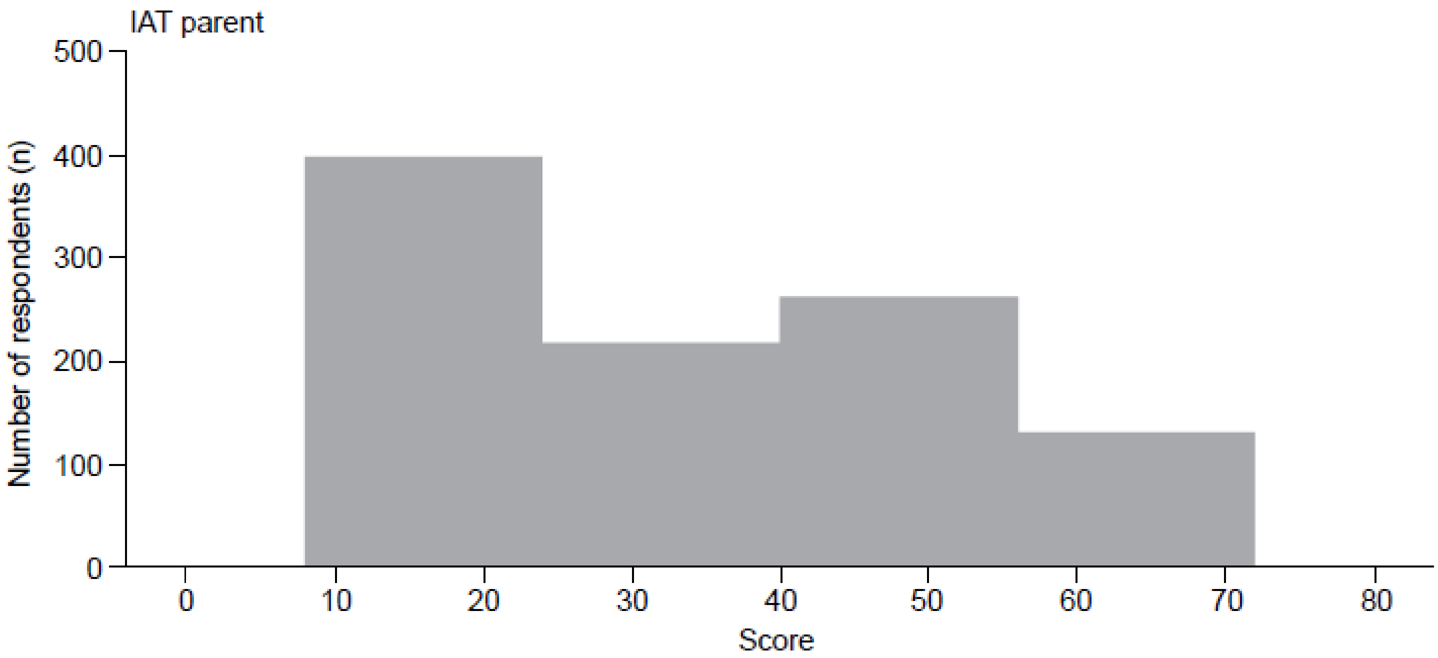

© 2023 Kimball HG et al. *JAMA Network Open*.

eFigure 3. Respondent Scores for the APQ Domains of Positive Parenting, Inconsistent Discipline, and Poor Monitoring and Supervision

a) APQ-SF Positive Parenting

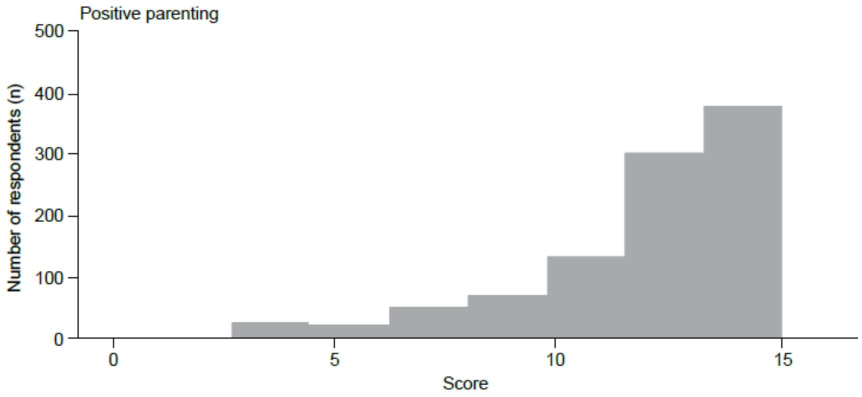

b) APQ-SF Inconsistent Discipline

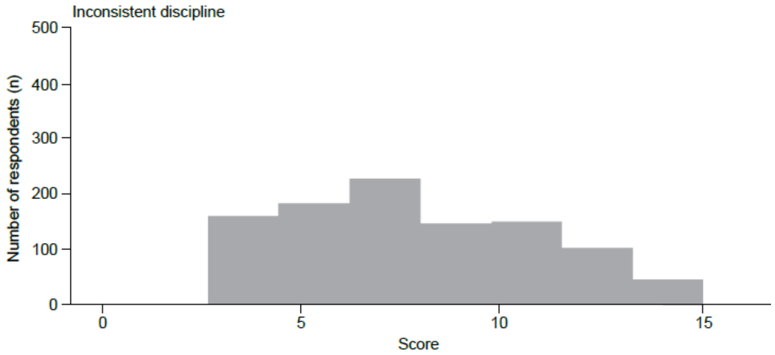

© 2023 Kimball HG et al. *JAMA Network Open*.

c) APQ-SF Poor Monitoring and Supervision

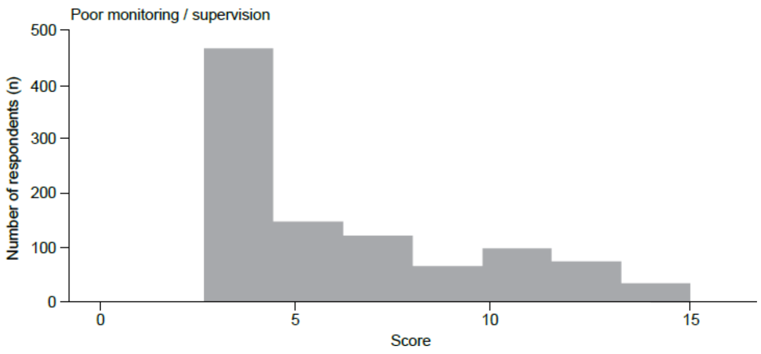

© 2023 Kimball HG et al. *JAMA Network Open*.

eFigure 4. Respondent Perception That Internet Use Increases Family Connectedness

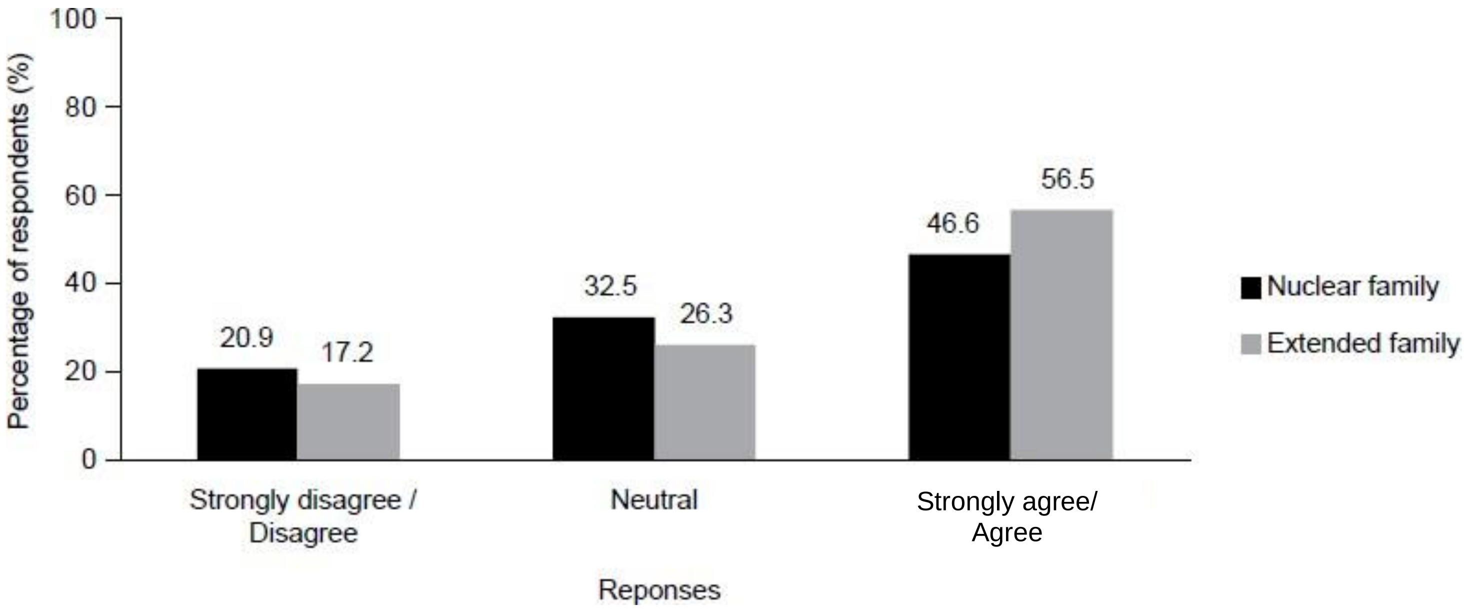

© 2023 Kimball HG et al. *JAMA Network Open*.

eFigure 5. Reasons Why the Internet Improves Family Connectedness and Mean Impact Score on the Family Due to Internet Use

a) Reasons for improvement

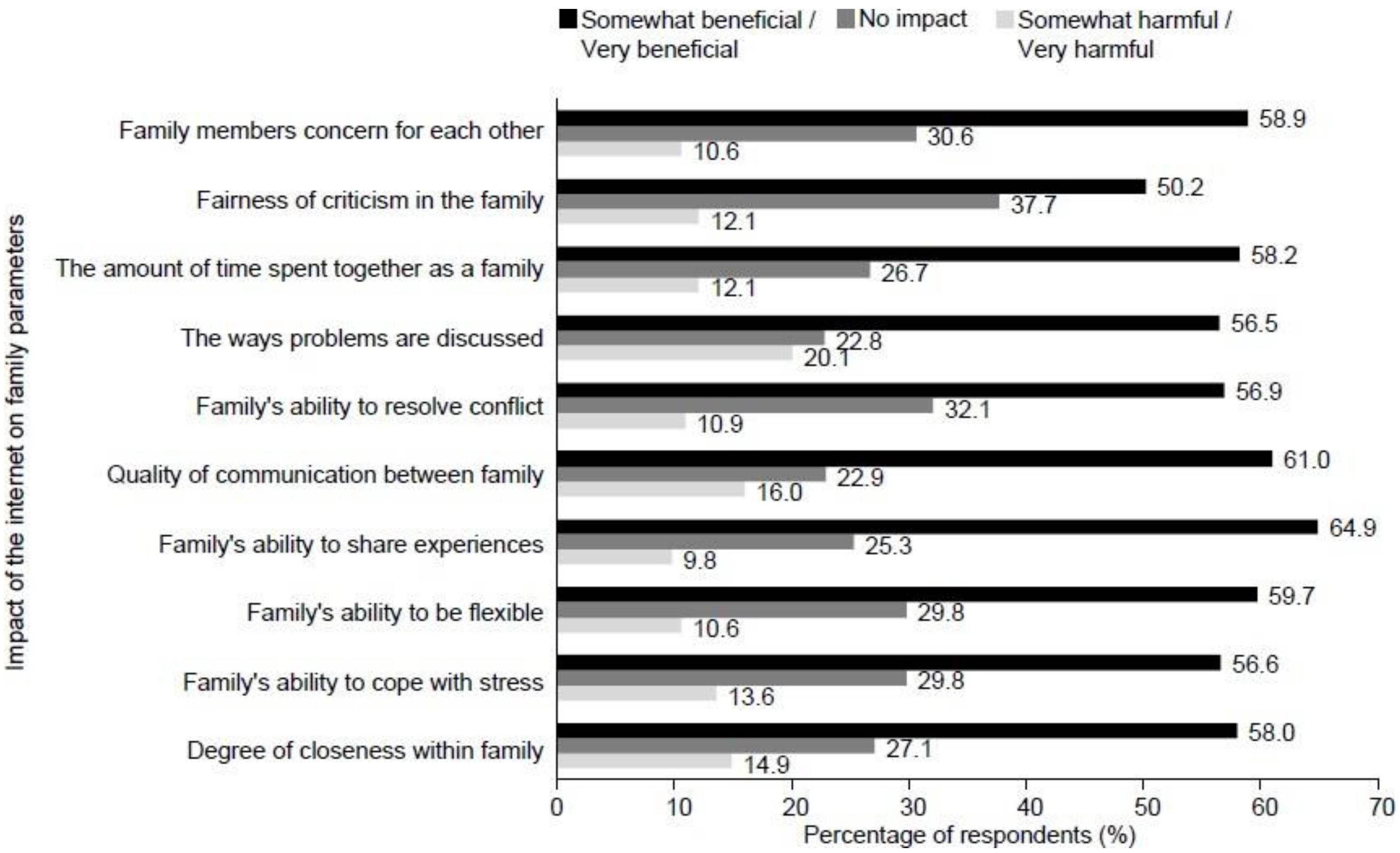

© 2023 Kimball HG et al. *JAMA Network Open*.

b) Mean family impact score

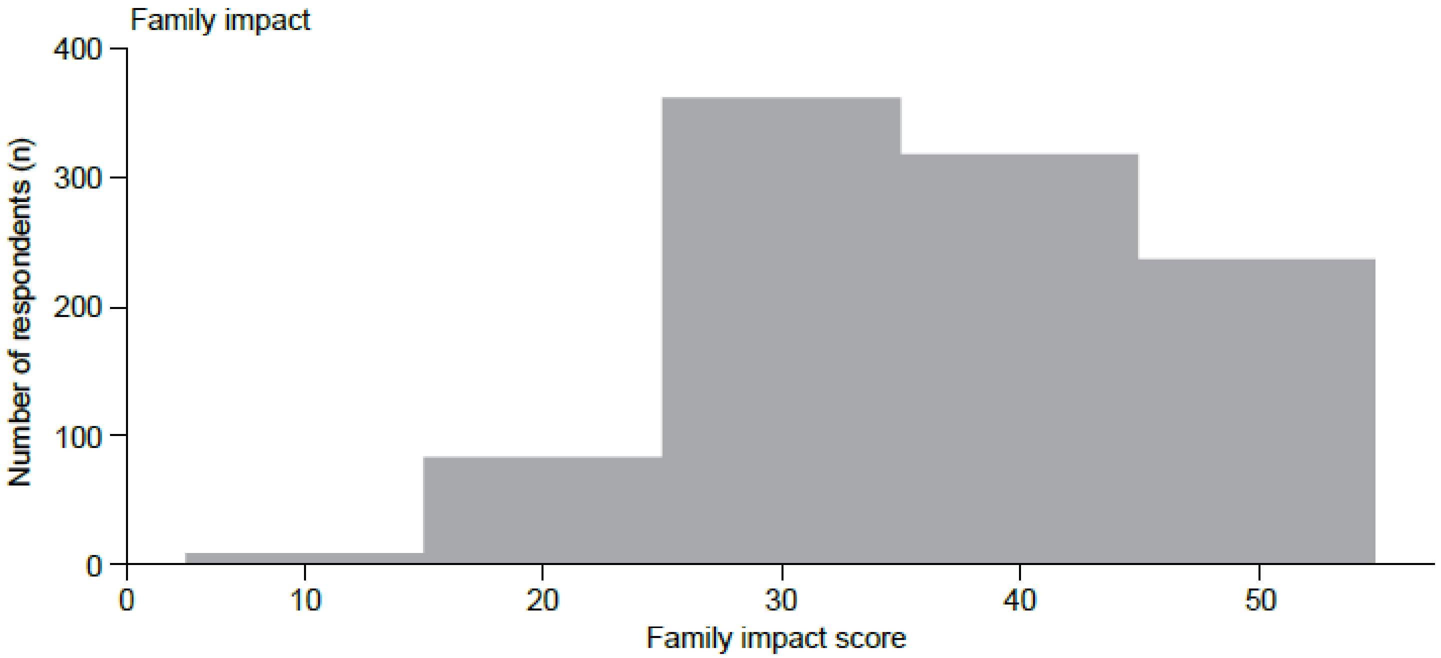

© 2023 Kimball HG et al. *JAMA Network Open*.

eFigure 6. Use of Specific Internet Platforms and Activities by Time

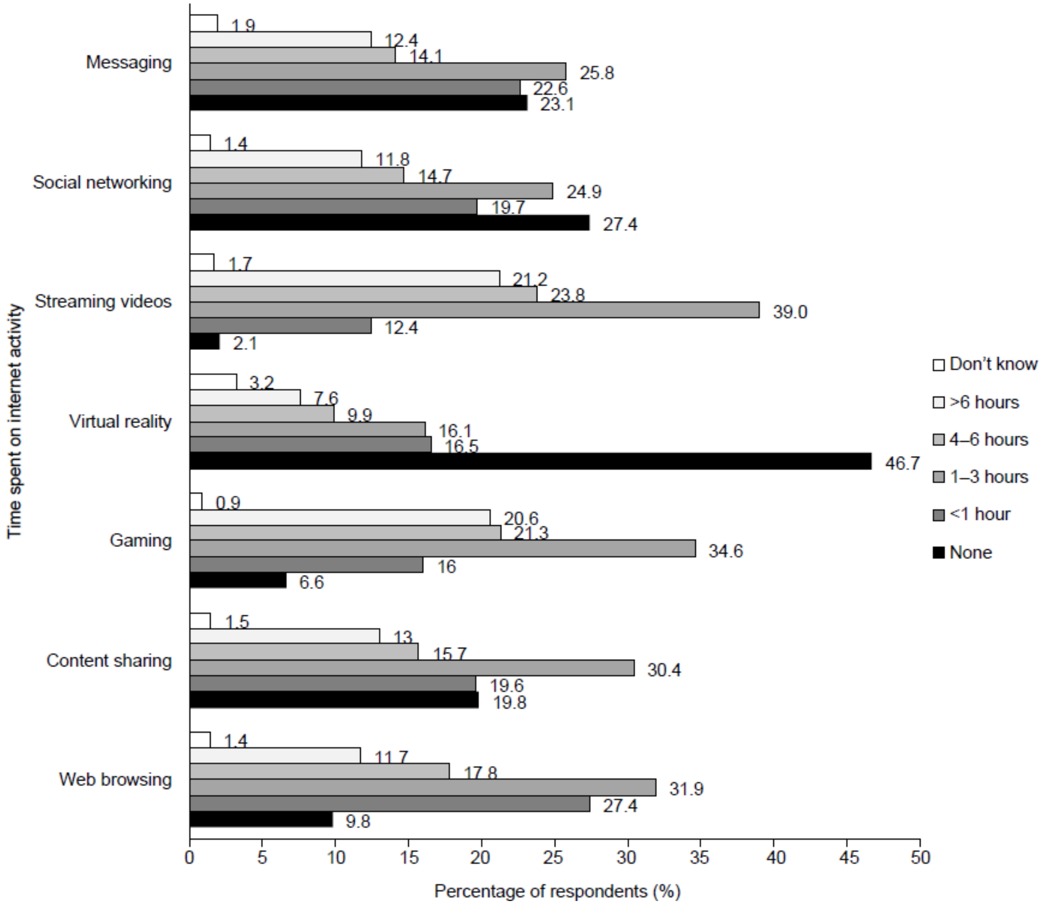

© 2023 Kimball HG et al. *JAMA Network Open*.
