# Supplementary material for: Parent-Perceived Benefits and Harms Associated With Internet Use by Adolescent Offspring
Source: JAMA Netw Open. 2023 Oct 26;6(10):e2339851. doi: 10.1001/jamanetworkopen.2023.39851 (PMC10603534; doi:10.1001/jamanetworkopen.2023.39851)
Supplement: Supplement 2. — Data Sharing Statement [file jamanetwopen-e2339851-s002.pdf]

Data Sharing Statement

Kimball. Parent-Perceived Benefits and Harms Associated With Internet Use by Adolescent Offspring. *JAMA Netw Open*. Published October 26, 2023.
doi:10.1001/jamanetworkopen.2023.39851

Data
Data available: Yes
Data types: Deidentified participant data
How to access data: lindsay.alexander@childmind.org
When available: With publication

Supporting Documents
Document types: None

Additional Information
Who can access the data: anyone requesting the data, researchers whose proposed use of the data has been approved
Types of analyses: anyone requesting the data, researchers whose proposed use of the data has been approved
Mechanisms of data availability: with investigator support
